# Triacylglycerols are preferentially oxidized over free fatty acids in heated soybean oil

**DOI:** 10.1038/s41538-021-00086-3

**Published:** 2021-04-01

**Authors:** Qing Shen, Zhichao Zhang, Shiva Emami, Jianchu Chen, Juliana Maria Leite Nobrega de Moura Bell, Ameer Y. Taha

**Affiliations:** 1 Department of Food Science and Technology, College of Agriculture and Environmental Sciences, University of California Davis, Davis, CA USA; 2 College of Biosystems Engineering and Food Science, Zhejiang University, Hangzhou, China; 3 Department of Biological and Agricultural Engineering, University of California Davis, Davis, CA USA

**Keywords:** Chemistry, Chemical biology

## Abstract

In oil, free fatty acids (FFAs) are thought to be the preferred substrate for lipid oxidation although triacylglycerols (TAGs) are the predominant lipid class. We determined the preferential oxidation substrate (TAGs versus FFAs) in soybean oil heated at 100 °C for 24 h, after validating a method for quantifying esterified and free lipid oxidation products (i.e., oxylipins) with mass-spectrometry. Reaction velocities and turnover (velocity per unit substrate) of FFA, and free and TAG-bound (esterified) oxylipins were determined. FFA hydrolysis rate and turnover were orders of magnitude greater (16-4217 fold) than that of esterified and free oxylipin formation. The velocity and turnover of TAG-bound oxylipins was significantly greater than free oxylipins by 282- and 3-fold, respectively. The results suggest that during heating, TAGs are preferentially oxidized over FFAs, despite the rapid hydrolysis and availability of individual FFAs as substrates for oxidation. TAG-bound oxylipins may serve as better markers of lipid oxidation.

## Introduction

In the US, soybean oil is the primary source of the essential polyunsaturated fatty acids (PUFAs)^1,2^, linoleic acid (LA; C18:2n-6) and α-linolenic acid (ALA; C18:3n-3)^3,4^. It is commonly used in food processing (i.e., frying, cooking), where the application of heat can oxidize PUFAs^5^. The oxidation of LA and ALA produces primary oxidized compounds known as oxylipins, which have been detected in various oils stored at room temperature^6^. Oxylipin concentrations have been shown to increase during the thermal processing of oil^7–9^.

**Fig. 1:**
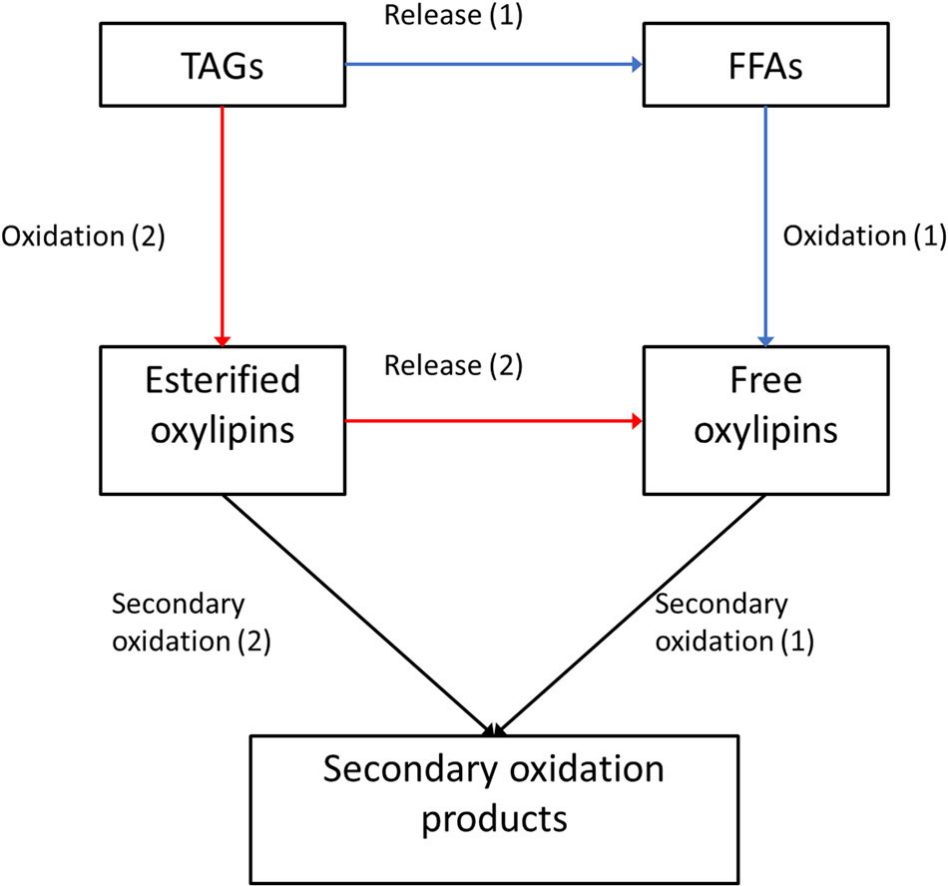
Pathway depiction of how free and esterified oxylipins could form in heated oil. TAGs release FFAs which undergo oxidation to form free oxylipins (Pathway 1, blue arrow). Alternatively, polyunsaturated fatty acids on TAGs can be directly oxidized to form esterified oxylipins (Pathway 2 in red). The oxidized oxylipins on TAGs can be released to generate free oxylipins (Pathway 2 in red). Secondary oxidation products can be generated by the breakdown of free or esterified oxylipins^32^. TAGs Triacylglycerols, FFAs free fatty acids.

In crude soybean oil, the main substrates for lipid oxidation are triacylglycerols (TAGs) and free fatty acids (FFAs), which make up 95–97% and 0.3–0.7% of oil, respectively^10,11^. Both TAGs and FFAs have been shown to oxidize during thermal treatment of oil^8,12–14^. As shown in Fig. 1, there are two plausible pathways through which these substrates produce oxylipins in heated oil—(1) TAGs can undergo non-enzymatic hydrolysis to produce FFAs, which can oxidize to form free oxylipins (Pathway 1 in blue), or (2) fatty acids within a TAG molecule (i.e. esterified fatty acids) can undergo oxidation to generate TAG-bound oxylipins (i.e. esterified oxylipins as shown in Pathway 2 in red). The TAGs containing bound oxylipins can then break down to generate free oxylipins (Pathway 2 in red). The non-enzymatic breakdown of free or esterified oxylipins can generate secondary oxidation products known as ‘secondary volatiles’.

Generally, the first mechanism involving FFA generation is presumed to be the primary cause of lipid oxidation, which is why percent FFA is routinely used as a marker of lipid oxidation^15–17^. Hence, more % FFA in a sample reflects more TAG hydrolysis into FFAs, and greater potential for the released FFAs to form primary oxidation products (i.e., oxylipins) and secondary volatiles. However, in oil, fatty acid hydroperoxides (a type of oxylipin) were detected in both TAGs and FFAs^8,12–14^, suggesting that oxidation can occur directly on TAGs, or on FFAs following their release from TAGs. The preferential substrate for lipid oxidation remains unknown.

In this study, we evaluated whether TAGs or FFAs are the preferred substrate for lipid oxidation in soybean oil. This was achieved by measuring the rate of free and esterified (mostly TAG) oxylipin formation in soybean oil heated for up to 24 h, after developing a method for separating and hydrolyzing esterified oxylipins in oil. The rate of FFA hydrolysis was also measured. Turnover, representing the rate of product formed per precursor substrate was calculated to determine whether observed rates were dependent on differing precursor substrate amounts (e.g., TAGs being more concentrated than FFAs in oil). We hypothesized that the rate of TAG oxylipin formation and turnover (i.e., reaction rate per unit substrate concentration) would be faster than that of FFAs due to the overwhelming abundance of TAGs as an oxidation substrate in oil. Below, we present evidence in support of the hypothesis.

## Results

### Experiment 1. Optimal soybean oil volume for measuring total oxylipins

Previously, total oxylipins (free + esterified) were determined by hydrolyzing 10 µL of oil with sodium carbonate (Na_2_CO_3_)^6^. However, the dependence of the hydrolysis reaction on oil volume has not been tested. It is possible that excess oil may reduce hydrolysis efficiency, leading to lower oxylipin values. To address this, concentrations of total LA- and ALA-derived oxylipins listed in Supplementary Table 1 were measured in different soybean oil volumes (1, 2, 5, and 10 μL) following Na_2_CO_3_ hydrolysis, using ultra high-pressure liquid chromatography-tandem mass spectrometry (UPLC-MS/MS). As shown in Table 1, one-way analysis of variance (ANOVA) followed by Tukey’s post-hoc test showed that oxylipin concentrations were significantly higher in 1 μL of soybean oil (~1 mg) compared to other volumes (*p* < 0.05). Oxylipin concentrations decreased with increasing oil volumes, suggesting inefficiencies in the hydrolysis reaction when more oil is present. Therefore, 1 μL of soybean oil (~1 mg) is the optimal oil volume for measuring total oxylipin concentrations with Na_2_CO_3_ hydrolysis, compared to 2, 5, and 10 µL.

**Table 1:** Concentrations of total linoleic acid (LA)-derived and α-linolenic acid (ALA)-derived oxylipins in different volumes of soybean oil (pmol/μL).

Volume of soybean oil (μL)	1	2	5	10	10 (ref. ^6^)	10 (ref. ^6^)
9-HODE	7.20 ± 0.93^a^	5.23 ± 0.92^b^	2.70 ± 0.08^c^	1.76 ± 0.11^c^	0.18(0.17, 0.23)	0.33 (0.26, 0.80)
13-HODE	12.13 ± 1.38^a^	7.40 ± 0.10^b^	4.74 ± 0.30^c^	2.87 ± 0.45^c^	0.85 (0.53, 0.86)	1.57 (1.03, 2.10)
9-oxo-ODE	8.90 ± 1.59^a^	5.18 ± 0.15^b^	3.11 ± 0.19^bc^	1.66 ± 0.20^c^	0.54 (0.26, 1.26)	0.89 (0.72, 1.59)
13-oxo-ODE	8.45 ± 1.32^a^	4.80 ± 0.75^b^	3.15 ± 0.57^bc^	2.09 ± 0.23^c^	0.07 (0.04, 0.12)	0.28 (0.15, 0.35)
9(10)-EpOME	10.15 ± 1.36^a^	6.83 ± 0.87^b^	3.61 ± 0.65^c^	1.48 ± 0.25^c^	20.50 (9.75, 40.55)	4.43 (3.14, 5.35)
12(13)-EpOME	21.12 ± 3.79^a^	16.46 ± 3.88^ab^	9.41 ± 1.10^bc^	4.02 ± 0.47^c^	35.15 (15.30, 56.00)	5.10 (3.51, 6.14)
9,10-DiHOME	20.00 ± 1.61^a^	15.08 ± 1.15^b^	11.83 ± 0.22^c^	8.30 ± 0.51^d^	15.60 (8.20, 19.75)	23.90 (14.20, 28.80)
12,13-DiHOME	18.79 ± 2.47^a^	14.57 ± 0.63^b^	11.53 ± 0.16^b^	7.90 ± 0.91^c^	10.25 (5.35, 13.85)	18.80 (10.87, 22.70)
9,10,13-TriHOME	NQ^1^	NQ	NQ	5.87 ± 7.01	NQ	NQ
9,12,13-TriHOME	NQ	NQ	NQ	6.65 ± 7.85^a^	NQ	NQ
ΣLA-derived oxylipins	106.76 ± 11.34^a^	75.55 ± 3.06^b^	50.09 ± 2.37^c^	42.59 ± 14.09^c^	83.14 (39.6, 132.62)	55.30 (33.88, 67.83)
9-HOTrE	0.93 ± 0.12^a^	0.56 ± 0.03^b^	0.36 ± 0.01^c^	0.24 ± 0.04^c^	0.05 (0.01, 0.05)	0.10 (0.06−0.14)
13-HOTrE	0.79 ± 0.06^a^	0.42 ± 0.01^b^	0.31 ± 0.02^c^	0.20 ± 0.02^d^	0.02 (0.02, 0.04)	0.16 (0.11−0.24)
ΣALA-derived oxylipins	1.72 ± 0.18^a^	0.98 ± 0.03^b^	0.67 ± 0.02^c^	0.44 ± 0.06^c^	0.07 (0.03, 0.09)	0.26 (0.17–0.38)

Data are expressed as mean ± SD of *n* = 3 per oil volume (1, 2, 5, and 10 µL).

^a-d^Different letters within a row are significantly different by one-way ANOVA followed by Tukey’s post-hoc test (*p* < 0.05).

^1^NQ: not quantified because the d4-prostaglandin E2 (d4-PGE2) surrogate standard used to quantify the analyte degraded during hydrolysis.

**Fig. 2:**
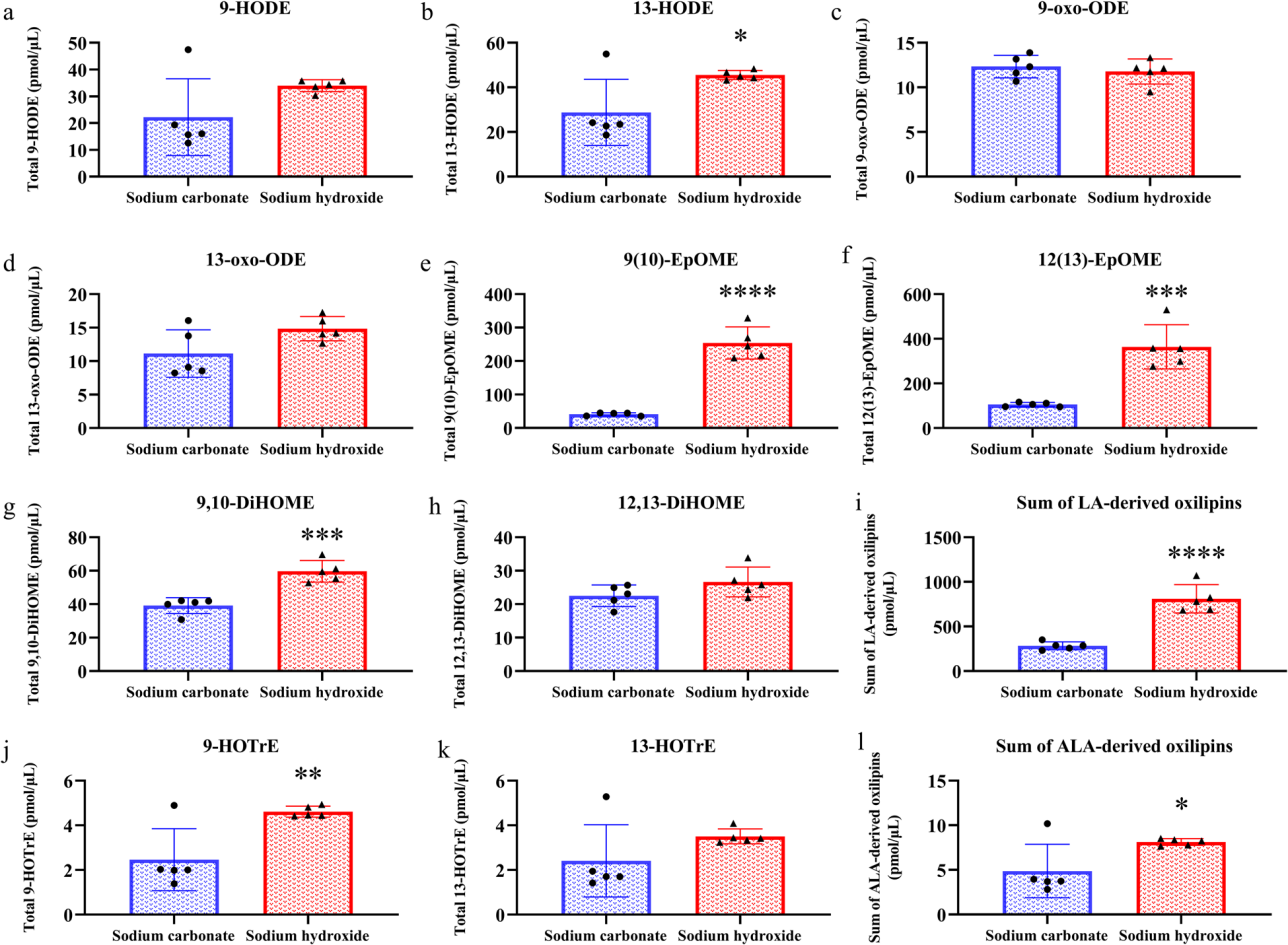
Concentrations of total linoleic acid (LA)-derived and α-linolenic acid (ALA)-derived oxylipins in 1 µL soybean oil hydrolyzed with sodium carbonate or sodium hydroxide. Data are expressed at mean ± SD of *n* = 5 per base type. *p < 0.05; **p < 0.01; ***p < 0.001; ****p < 0.0001 by unpaired *t*-test.

^2^Reference data from Fig. 2 (Experiment 1) in Richardson et al. (2017). Concentrations are expressed as median and interquartile range (25th and 75th percentiles).

^3^Reference data from Table 2 (Experiment 2) in Richardson et al. (2017). Concentrations are expressed as median and interquartile range (25th and 75th percentiles).

The last two columns of Table 1 show published oxylipin concentrations in soybean oil from our previous study, which measured oxylipins in 10 μL oil following hydrolysis with Na_2_CO_3_^6^. In retrospect, this oil volume was too high (based on our current findings). However, concentrations of most oxylipins were comparable to our current results in 10 μL oil, reflecting the reproducibility of our assays. The only exception was 9(10)-epoxyoctadecamonoenoic acid (9(10)-EpOME), which was 2 to 27 times lower in this study (1.48 pmol/μL) compared to our previous study (3.14–40.55 pmol/µL), suggesting possible instability in soybean oil^6^.

Matrix effects and surrogate percent recovery were determined to test whether the lower oxylipin concentrations achieved with higher oil volumes were due to ion suppression or enhancement, or to changes in the surrogate recovery during solid phase extraction (SPE). As shown in Supplementary Table 2, matrix effects were minimal across all oil volumes and ranged between 80–120% for most metabolites. The percent surrogate recovery shown in Supplementary Fig. 1 was generally lower in the 5 and 10 µL oil samples compared to 1 and 2 µL oil. Notably, the low surrogate standard recovery at higher oil volumes (5 µL and 10 µL) was associated with proportional reductions in analyte peak areas, suggesting that both the surrogate standards and the analytes they quantify, had poor recoveries at higher oil volumes. Thus, overall, niether matrix effects nor surrogate standard recoveries explained for the differences in concentrations between the different oil volumes. As mentioned above, inefficiencies in the hydrolysis at higher oil volumes likely explain the observed reductions in oil oxylipins concentrations at 2, 5, and 10 µL, compared to 1 µL.

### Experiment 2. Comparing the hydrolysis efficiency of different bases

The hydrolysis efficiency of Na_2_CO_3_ and sodium hydroxide (NaOH) on 1 µL soybean oil was compared, in view of recent studies showing NaOH efficiently hydrolyses esterified oxylipins in human plasma^18,19^. As shown in Fig. 2, unpaired t-test analysis revealed that LA-derived 13-hydroxyoctadecadienoic acid (13-HODE), 12(13)-epoxyoctadecamonoenoic acid (12(13)-EpOME), 9(10)-EpOME, and 9,10-dihydroxyoctadecamonoenoic acid (9,10-DiHOME), and ALA-derived 9-hydroxyoctadecatrienoic acid (9-HOTrE) concentrations were significantly higher by 18% to 521% with NaOH hydrolysis compared to Na_2_CO_3_ hydrolysis. Hence, the sum of LA-derived and ALA-derived oxylipins was significantly higher by 187% and 67%, respectively, following NaOH hydrolysis compared to Na_2_CO_3_ hydrolysis.

Minimal matrix effects were observed following hydrolysis with either base (Supplementary Table 3). Surrogate percent recoveries for d-11-11(12)-epoxyeicosatrienoic acid (d-11-11(12)-EpETrE), d11-14,15-dihydroxyeicosatrienoic acid (d11-14,15-DiHETrE), d4-9-hydroxyoctadecadienoic acid (d4-9-HODE), d4-leukotriene B4 (d4-LTB4), d6-20-hydroxyeicosatetraenoic acid (d6-20-HETE), and d8-5-hydroxyeicosatetraenoic acid (d8-5-HETE) were significantly higher (by 15 to 216%) with NaOH compared to Na_2_CO_3_ (Supplementary Fig. 2), owing to the greater peak intensity in the NaOH-hydrolyzed samples compared to Na_2_CO_3_. Higher peak intensities were also observed for the analytes quantified by these surrogates. The recovery of d4-thromboxane B2 (d4-TXB2) was 47% lower with NaOH compared to Na_2_CO_3_ treated samples, but d4-TXB2 was not used for quantifying LA and ALA metabolites. Overall, the data suggest that NaOH is more efficient at hydrolyzing bound oxylipins than Na_2_CO_3_.

### Experiment 3. Optimal oil volume needed to separate free oxylipins with SPE

**Experiment 3** tested the appropriate soybean oil volume (1, 2, 5, or 8 µL) needed to separate free from esterified oxylipins using a silica column (100 mg; Waters, Milford, MA; Cat #WAT023595), which selectively traps polar lipids such as free oxylipins, and elutes non-polar TAGs. As shown in Table 2, free 9-HODE, 13-HODE, 9-oxo-octadecadienoic acid (9-oxo-ODE), 12(13)-EpOME, and 9,10-DiHOME were significantly lower in the 2, 5, and 8 µL oil, compared to 1 µL oil (*p* < 0.05 by one-way ANOVA). As noted in the table legend, there was unexpected background noise originating from the blank extract (See Methods) for 13-HODE and 9-oxo-ODE, and therefore changes in these two compounds should be interpreted with caution. Free 9(10)-EpOME concentration did not significantly differ between oil volumes. LA-derived 9,10,13-trihydroxyoctadecamonoenoic acid (9,10,13-TriHOME) was only detected at 2 µL oil. ALA-derived 9-HOTrE was detected at 5 µL and 8 µL, but not lower volumes. The sum of free LA-derived oxylipins was significantly higher in 1 µL oil compared to 2, 5, and 8 µL (*p* < 0.05).

**Table 2:** Concentrations of free linoleic acid (LA)-derived and α-linolenic acid (ALA)-derived oxylipins in different volumes of soybean oil (pmol/μL).

Volume of soybean oil (μL)	1	2	5	8
9-HODE	0.37 ± 0.02^a^	0.19 ± 0.02^b^	0.10 ± 0.01^c^	0.09 ± 0.02^c^
13-HODE	0.55 ± 0.04^a^	0.33 ± 0.06^b^	0.18 ± 0.02^c^	0.17 ± 0.01^c^
9-oxo-ODE	0.32 ± 0.04^a^	0.22 ± 0.02^b^	0.11 ± 0.01^c^	0.09 ± 0.01^c^
13-oxo-ODE	ND^1^	ND	ND	ND
9(10)-EpOME	0.11 ± 0.10^2^	0.13 ± 0.01	0.10 ± 0.004	0.08 ± 0.01
12(13)-EpOME	0.45 ± 0.06^a^	0.34 ± 0.03^b^	0.30 ± 0.02^b^	0.29 ± 0.02^b^
9,10-DiHOME	0.16 ± 0.01^a^	0.12 ± 0.01^b^	0.10 ± 0.01^b^	0.10 ± 0.003^b^
12,13-DiHOME	0.12 ± 0.003^a^	0.10 ± 0.02^ab^	0.09 ± 0.004^b^	0.09 ± 0.004^b^
9,10,13-TriHOME	ND	0.06 ± 0.08^2^	ND	ND
9,12,13-TriHOME	ND	ND	ND	ND
ΣLA-derived oxylipins	2.08 ± 0.08^a^	1.49 ± 0.20^b^	0.98 ± 0.05^c^	0.91 ± 0.02^c^
9-HOTrE	ND	ND	0.01 ± 0.004	0.01 ± 0.003
13-HOTrE	ND	ND	ND	ND
ΣALA-derived oxylipins	NA^1^	NA	0.01 ± 0.004	0.01 ± 0.003

Data are expressed as mean ± SD of *n* = 3 per oil volume. In this particular experiment, the peak areas of free 13-HODE and 9-oxo-ODE in the solvent blank extract (*n* = 1) accounted for 25–69% and 35–108% of the peak areas detected in oil samples, respectively, suggesting background contamination from the silica or HLB columns used to purify the separated oxylipins. We estimated % background contribution in each sample, using the blank peak area. For 13-HODE, the background peak accounted for 66.2 ± 4.4, 56.4 ± 9.2, 41.5 ± 3.6, and 26.2 ± 0.8% of free oxylipins in 1, 2, 5, and 8 µL oil volumes, respectively. For 9-oxo-ODE, the background peak accounted for 93.9 ± 12.3, 66.9 ± 5.0, 53.7 ± 5.6, and 41.6 ± 5.6% of free oxylipins in 1, 2, 5, and 8 µL oil volumes, respectively.

^1^ND: not detected, or NA: non-applicable

^2^One of the three samples was not detected, so it was imputed with LOQ/sqrt(2).

^a–c^Different letters within a row are significantly different by one-way ANOVA followed by Tukey’s post-hoc test (*p* < 0.05).

The matrix effects were minimal across oil volumes, as shown in Supplementary Table 4. There were no significant differences in deuterated surrogate recoveries amongst the different volumes (Supplementary Fig. 3). Thus, matrix effects and surrogate recoveries did not explain the differences in concentrations between the different oil volumes. In addition, no free oxylipins were found in the neutral lipid fraction, suggesting that free oxylipins were well-separated from TAGs using the silica SPE columns. As shown in Supplementary Table 5, which embeds the raw chromatograms, free deuterated standards added to the oil samples were only seen in the polar/free fraction, but not the neutral lipid fraction containing TAG-bound oxylipins (across all volumes). This confirms that only free oxylipins were retained in the silica column, and TAG-bound oxylipins were eluted from the column. The observed reduction in concentrations at higher volumes are likely due to column overloading, which could make it difficult for free oxylipins to completely elute from the silica column during the methanol wash. Overall, our analysis demonstrates that 1 µL oil (~1 mg) is the ideal volume to apply onto 100 mg silica SPE column, to separate free from bound oxylipins.

### Experiment 4. Effect of heating on fatty acid and oxylipin concentrations

The change in temperature over time is shown in Supplementary Fig. 4. Oil temperature reached 100 °C within 10 min of being on a heating block maintained at 100 °C, and was stable throughout the 24 h period. Oil sampled during this period was subjected to total and free fatty acid and oxylipin analysis. For oxylipins, only 1 µL was analyzed, as this volume yielded the highest amount of total and free oxylipins compared to higher volumes (based on **Experiments 1**–**3**).

Table 3 presents fatty acids within total (i.e., free + esterified) and free lipid pools during the 24 h heating period. One-way repeated measures ANOVA showed that fatty acids within total lipids did not change significantly over time compared to baseline (Table 3). However, free palmitic acid, stearic acid, oleic acid, LA and ALA increased significantly by 1.3 to 2.4-fold at 24 h compared to baseline (0 h), 4 or 8 h (*p* < 0.05). This was reflected in the sum of FFAs, which increased significantly by approximately 2-fold at 24 h (*p* < 0.05). FFAs accounted for less than 1% of total fatty acids during the 0–8 h of heating period. At 24 h, FFAs significantly increased to 1.6% of total fatty acids (*p* < 0.05, Table 3).

**Table 3:** Concentrations of total fatty acids (free + esterified) and free fatty acids in soybean oil heated for 24 h (nmol/μL).

Heating time (h)	0	1	4	8	24
Total palmitic acid	316.68 ± 12.99^ab^	310.33 ± 20.06^a^	336.72 ± 21.07^ab^	330.66 ± 28.50^b^	352.07 ± 21.16^b^
Total stearic acid	110.51 ± 4.88^ab^	108.42 ± 6.92^a^	117.52 ± 7.32^ab^	115.63 ± 9.55^b^	123.14 ± 7.46^b^
Total oleic acid	644.27 ± 23.47^ab^	630.03 ± 42.63^a^	687.36 ± 43.26^ab^	670.45 ± 61.23^b^	717.84 ± 43.29^b^
Total linoleic acid (LA)	1432.76 ± 52.54^ab^	1396.88 ± 89.91^a^	1513.97 ± 96.25^ab^	1486 ± 131.21^b^	1567.73 ± 96.44^b^
Total linolenic acid (ALA)	174.74 ± 6.20^ab^	170.35 ± 10.96^a^	184.61 ± 11.86^ab^	180.9 ± 16.29^b^	188.98 ± 11.74^b^
Σ Total fatty acids	2678.95 ± 99.67	2616.02 ± 170.39	2840.19 ± 179.72	2783.65 ± 246.73	2949.75 ± 179.88
Free palmitic acid	5.77 ± 2.84^ab^	3.96 ± 0.57^a^	4.00 ± 0.49^a^	4.56 ± 1.02^ab^	7.47 ± 1.27^b^
Free stearic acid	1.57 ± 0.26^a^	1.56 ± 0.18^a^	1.54 ± 0.22^ab^	1.59 ± 0.13^ab^	2.66 ± 0.45^b^
Free oleic acid	4.92 ± 0.73^a^	5.00 ± 0.23^a^	5.1 ± 0.34^a^	5.40 ± 0.71^ab^	10.06 ± 1.88^b^
Free linoleic acid (LA)	10.01 ± 0.63^a^	11.24 ± 0.66^ab^	11.6 ± 0.59^b^	12.75 ± 1.60^ab^	24.07 ± 3.52^c^
Free linolenic acid (ALA)	1.28 ± 0.18^a^	1.49 ± 0.15^a^	1.53 ± 0.20^ab^	1.62 ± 0.26^a^	2.91 ± 0.39^c^
Σ Free fatty acids	23.55 ± 2.80^a^	23.24 ± 0.97^a^	23.78 ± 1.38^a^	25.92 ± 3.07^a^	47.16 ± 7.46^b^
%Free fatty acids	0.88 ± 0.001^a^	0.89 ± 0.09^a^	0.84 ± 0.04^a^	0.93 ± 0.05^a^	1.60 ± 0.25^b^

Data are expressed at mean ± SD of *n* = 5 per time-point.

^a–c^Different letters within a row are significantly different by repeated measures one-way ANOVA followed by Tukey’s post-hoc test (*p* < 0.05).

Table 4 shows the concentrations of total (i.e., free + esterified) and free LA-derived and ALA-derived oxylipins in soybean oil at baseline (0 h) and at 1, 4, 8, and 24 h of heating at 100 °C. Total oxylipin concentrations were 339 to 440 times greater than free oxylipins (Table 4). In the total oxylipin pool, LA-derived oxylipins were 30–105 times greater in concentration than the two ALA-derived oxylipins measured. In the free pool, ALA-derived oxylipins were not detected at any time point.

**Table 4:** Total (free + esterified) and free oxylipin concentrations in soybean oil heated for 24 h (pmol/μL).

Heating time (h)	0	1	4	8	24
Total 9-HODE	52.77 ± 6.76^a^	50.84 ± 4.98^a^	65.44 ± 9.88^a^	88.53 ± 12.16^b^	235.56 ± 71.02^b^
Total 13-HODE	61.68 ± 8.65^a^	62.44 ± 6.12^a^	76.03 ± 12.00^a^	98.37 ± 12.33^b^	411.24 ± 178.99^ab^
Total 9-oxo-ODE	17.24 ± 0.82^a^	25.99 ± 3.06^b^	37.71 ± 3.35^c^	56.52 ± 7.02^d^	94.95 ± 39.48^abcd^
Total 13-oxo-ODE	28.21 ± 1.56^a^	31.64 ± 4.53^a^	39.69 ± 4.54^b^	54.44 ± 7.98^c^	75.44 ± 27.34^abc^
Total 9(10)-EpOME	348.97 ± 50.30	315.92 ± 40.90	297.93 ± 50.45	355.66 ± 57.07	380.26 ± 136.88
Total 12(13)-EpOME	537.90 ± 55.91	496.95 ± 69.71	477.81 ± 66.46	556.23 ± 87.97	642.86 ± 199.27
Total 9,10-DiHOME	64.06 ± 2.31	60.53 ± 3.83	64.46 ± 7.37	66.77 ± 7.53	62.37 ± 20.60
Total 12,13-DiHOME	25.74 ± 1.09	24.69 ± 2.01	26.58 ± 3.61	28.06 ± 4.05	33.12 ± 10.00
Total 9,10,13-TriHOME	NQ^1^	NQ	NQ	NQ	NQ
Total 9,12,13-TriHOME	NQ	NQ	NQ	NQ	NQ
Σ Total LA-derived oxylipins	1136.57 ± 101.43^a^	1069 ± 115.67^a^	1085.64 ± 134.95^a^	1304.58 ± 154.4^ab^	1935.8 ± 299.23^b^
Total 9-HOTrE	6.37 ± 0.87^ab^	5.78 ± 0.51^a^	6.63 ± 1.06^ab^	7.79 ± 0.88^b^	22.39 ± 8.74^ab^
Total 13-HOTrE	4.43 ± 0.75^a^	4.93 ± 0.59^a^	6.58 ± 0.81^b^	9.14 ± 1.16^c^	41.54 ± 24.51^abc^
Σ Total ALA-derived oxylipins	10.79 ± 1.57^a^	10.70 ± 0.99^a^	13.21 ± 1.81^a^	16.94 ± 1.97^b^	63.93 ± 33.18^ab^
Free 9-HODE	0.37 ± 0.19	0.39 ± 0.10	0.43 ± 0.18	0.40 ± 0.08	0.50 ± 0.11
Free 13-HODE	0.61 ± 0.24	0.58 ± 0.14	0.80 ± 0.28	0.81 ± 0.17	1.54 ± 0.64
Free 9-oxo-ODE	0.39 ± 0.06	0.38 ± 0.10	0.42 ± 0.03	0.43 ± 0.07	0.57 ± 0.14
Free 13-oxo-ODE	ND^2^	ND	ND	ND	ND
Free 9(10)-EpOME	0.18 ± 0.07	0.19 ± 0.02	0.21 ± 0.04	0.39 ± 0.35	0.51 ± 0.19
Free 12(13)-EpOME	0.39 ± 0.08^a^	0.45 ± 0.03^a^	0.52 ± 0.06^a^	0.98 ± 0.78^ab^	2.30 ± 0.67^b^
Free 9,10-DiHOME	0.30 ± 0.28	0.24 ± 0.05	0.27 ± 0.13	0.19 ± 0.04	0.18 ± 0.05
Free 12,13-DiHOME	0.24 ± 0.18	0.20 ± 0.01	0.24 ± 0.08	0.19 ± 0.05	0.16 ± 0.03
Free 9,10,13-TriHOME	0.12 ± 0.05	0.14 ± 0.03	0.14 ± 0.05	0.11 ± 0.02	0.11 ± 0.03
Free 9,12,13-TriHOME	ND	ND	ND	ND	ND
Σ Free LA-derived oxylipins	2.60 ± 1.11^ab^	2.57 ± 0.4^ab^	3.04 ± 0.73^ab^	3.50 ± 1.03^a^	5.86 ± 1.85^b^
Free 9-HOTrE	ND	ND	ND	ND	ND
Free 13-HOTrE	ND	ND	ND	ND	ND
Σ Free ALA-derived oxylipins	NA^2^	NA	NA	NA	NA
%Free oxylipins	0.23 ± 0.11	0.24 ± 0.05	0.28 ± 0.08	0.27 ± 0.10	0.30 ± 0.11

Data are expressed at mean ± SD of *n* = 5 per time-point.

^1^NQ: not quantified because the d4-PGE2 surrogate standard used to quantify the analyte degraded during hydrolysis.

^2^ND: not detected, or NA: non-applicable.

^a–d^Different letters within a row are significantly different by repeated-measures one-way ANOVA followed by Tukey’s post-hoc test (*p* < 0.05). *LA* linoleic acid, *ALA* α-linolenic acid.

One-way repeated measures ANOVA showed that heat increased the concentration of several oxylipins within the total oxylipin pool over time (Table 4). 9-HODE was significantly higher by 135–463% at 8 and 24 h compared to baseline, 1 and 4 h (*p* < 0.05). 13-HODE was significantly higher by 129–159% at 8 h compared to 0, 1, and 4 h (*p* < 0.05); values at 24 h were higher by 418–667% but non-significant compared to other time-points. 13-oxo-ODE was significantly higher at 8 h compared to prior time-points, and at 4 h compared to 0 and 1 h (*p* < 0.05). It was also higher at 24 h compared to baseline (by 267%) but the difference was not statistically significant. The same trend was observed for 9-oxo-ODE, except that its concentration at 1 h was significantly higher than baseline (*p* < 0.05). ALA-derived 13-HOTrE was higher by 133–149% at 4 h compared to 0 and 1 h, and at 8 h compared to prior time-points. The individual changes in LA-derived and ALA-derived oxylipins was reflected in the sums, which were significantly higher by 128–597% at 8 and 24 h compared to other time-points (*p* < 0.05).

In the free pool, 12(13)-EpOME concentration increased significantly by approximately 6-fold at 24 h compared to baseline, 1 and 4 h (*p* < 0.05). The sum of LA-metabolites was significantly higher by 169% at 24 h compared 8 h (*p* < 0.05). The sum of LA metabolites was also higher at 24 h compared to 0, 1, and 4 h (by 194–230%) but the difference was not statistically significant. Free ALA-metabolites were not detected at baseline or during heating. Free oxylipins constituted 0.2–0.3% of the total oxylipin pool, and did not differ significantly between time-points.

### Experiment 4. Kinetics of FFA hydrolysis and of LA-derived and ALA-derived oxylipin formation in soybean oil heated for 24 h

**Fig. 3:**
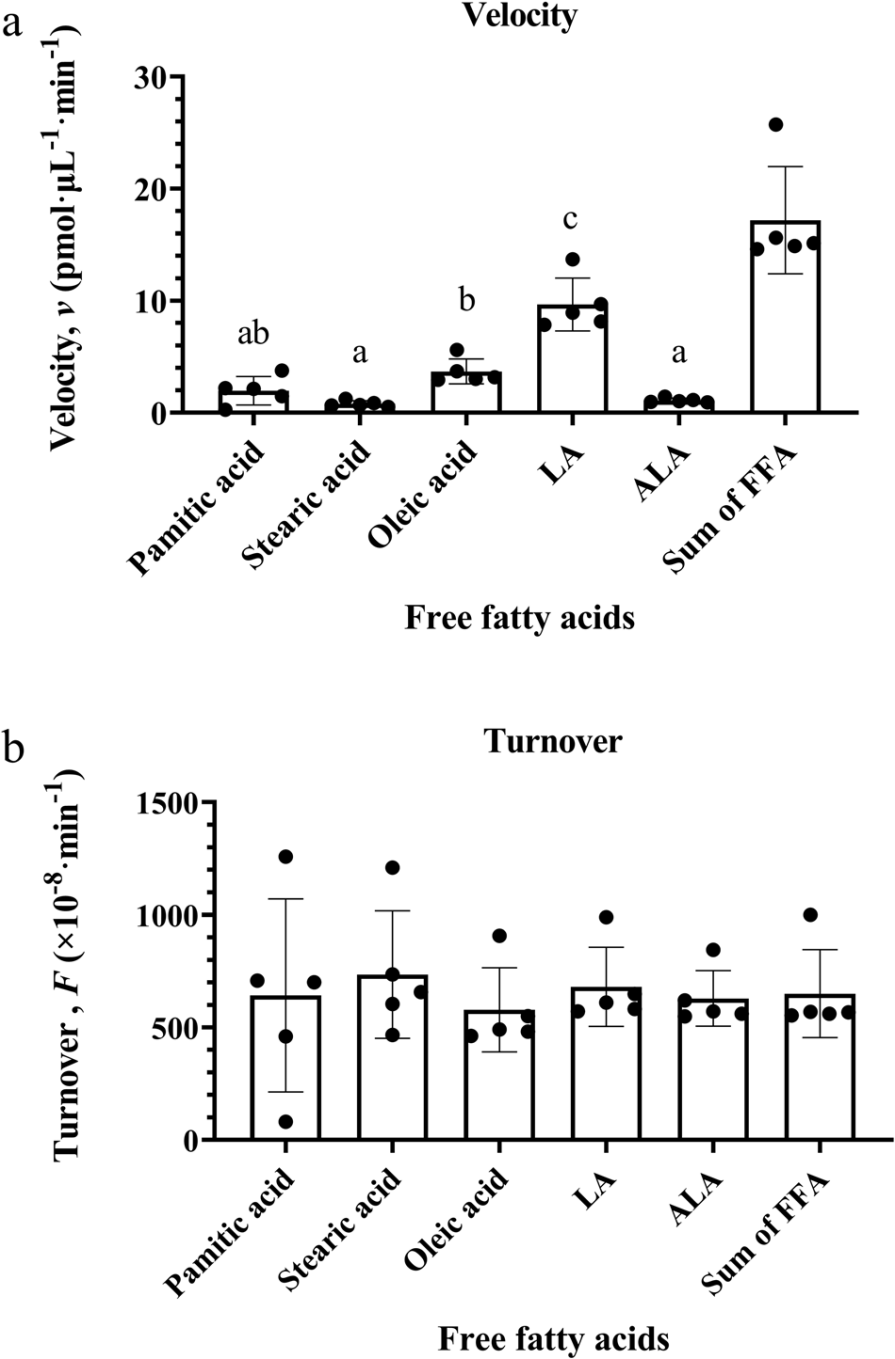
Velocity and turnover of free palmitic acid, stearic acid, oleic acid, linoleic acid (LA), and α-linolenic acid (ALA) hydrolysis in soybean oil heated for 24 h. Velocity (**a**) and turnover (**b**) data are expressed at mean ± SD of *n* = 5 per fatty acid. Different superscript letters are significantly different by one-way ANOVA followed by Tukey’s post-hoc test (*p* < 0.05). FFA, free fatty acid.

Figure 3 and Table 5 present the kinetics (reaction velocity and turnover) of FFA hydrolysis and oxylipin (total, esterified and free) formation, respectively.

**Table 5:** Velocity (*v*, pmol·μL^−1^·min^−1^) and turnover (*F*, ×10^–8^·min^−1^) of total, free and esterified oxylipins in soybean oil heated for 24 h.

Oxylipins	*v* (Total)	*v* (Esterified)	*v* (Free)	*F* (Total)	*F* (Esterified)	*F* (Free)
9-HODE	0.13 ± 0.054	0.13 ± 0.05	0.0001 ± 0.0001*	9.12 ± 3.48	9.17 ± 3.49	0.85 ± 1.24*
13-HODE	0.25 ± 0.13	0.25 ± 0.13	0.0006 ± 0.0005*	17.53 ± 9.22	17.60 ± 9.25	6.37 ± 4.69
9-oxo-ODE	0.052 ± 0.028	0.052 ± 0.028	0.0001 ± 0.0001*	3.63 ± 1.93	3.64 ± 1.94	1.33 ± 1.14
13-oxo-ODE	0.032 ± 0.019	0.032 ± 0.019	NQ ^1^	2.25 ± 1.29	2.27 ± 1.30	NQ
9(10)-EpOME	0.040 ± 0.082	0.039 ± 0.082	0.0002 ± 0.0002	2.69 ± 5.45	2.70 ± 5.49	2.18 ± 1.56
12(13)-EpOME	0.097 ± 0.14	0.095 ± 0.14	0.0014 ± 0.0005	6.61 ± 9.53	6.56 ± 9.62	13.74 ± 4.74
9,10-DiHOME	−0.0002 ± 0.014	−0.0002 ± 0.014	−0.0001 ± 0.0001	−0.0002 ± 0.0094	−0.0001 ± 0.0095	−0.0065 ± 0.0091
12,13-DiHOME	0.0056 ± 0.0069	0.0056 ± 0.0069	−0.0001 ± 0.0001	0.39 ± 0.49	0.40 ± 0.49	−0.0058 ± 0.0082
9,10,13-TriHOME	NQ	NQ	−0.00002 ± 0.00002	NQ	NQ	−0.0017 ± 0.0024
9,12,13-TriHOME	NQ	NQ	NQ	NQ	NQ	NQ
ΣLA-derived oxylipins	0.61 ± 0.20	0.61 ± 0.20	0.0023 ± 0.0015*	42.20 ± 12.38	42.33 ± 12.47	23.31 ± 14.74
9-HOTrE	0.012 ± 0.0067	0.012 ± 0.0067	NQ	6.65 ± 3.62	6.70 ± 3.63	NQ
13-HOTrE	0.027 ± 0.018	0.027 ± 0.018	NQ	15.01 ± 9.85	15.12 ± 9.90	NQ
ΣALA-derived oxylipins	0.038 ± 0.025	0.038 ± 0.025	NQ	21.65 ± 13.45	21.81 ± 13.51	NQ

Velocity and turnover data are expressed at mean ± SD of *n* = 5 per oxylipin within each lipid fraction (total, esterified and free).

^1^NQ: not quantified because the PGE2 surrogate standard used to quantify the analyte degraded during hydrolysis (for esterified TriHOMEs), or a slope was not determined from the Concentration-Time plot due to non-detected values over time (free 13-oxo-ODE, 9,12,13-TriHOME, 9-HOTrE, and 13-HOTrE).

*Velocity or turnover of esterified oxylipin is significantly different from that of free oxylipin (*p* < 0.05) by unpaired *t*-test.

The velocity and turnover of FFAs was determined from the linear fit of the Concentration-Time plots shown in Supplementary Fig. 5. Velocity, representing the rate of hydrolysis, was derived from the slope of the linear fit, and normalized to precursor esterified fatty acid concentration to obtain turnover (i.e., the rate of hydrolysis per unit substrate). One-way ANOVA followed by Tukey’s post-hoc test was used to compare the velocity of hydrolysis and turnover of the 5 major FFAs detected in soybean oil. As shown in Fig. 3, the rate of LA hydrolysis was significantly greater than the rate of palmitic acid, stearic acid, oleic acid and ALA hydrolysis by 4.9, 12.1, 2.6, and 8.8-fold, respectively (*p* < 0.05). The rate of oleic acid hydrolysis was significantly greater than that of stearic acid and ALA by 4.6-fold and 3.4-fold, respectively (*p* < 0.05), but did not significantly differ from that of palmitic acid. One-way ANOVA showed no significant differences in turnover between the various fatty acids, suggesting that per unit substrate, all fatty acids were hydrolyzed from TAGs at a similar rate.

Similar kinetics were applied to total and free oxylipins as shown in the Concentration-Time plots of Supplementary Fig. 6. The figure also shows the linear fit for esterified oxylipins, determined by subtracting the concentration of free from total oxylipins. Table 5 shows the rate of total, esterified and free oxylipin formation or disappearance derived from the slope of the linear fit, and turnover, determined by normalizing these rates to precursor fatty acid concentrations. A positive slope (rate) means more product formation over time, whereas a negative slope reflects more product degradation over time.

As expected, the rate of total oxylipin formation was similar in value to the rate of esterified oxylipins, since esterified oxylipins constituted the majority (>99.7%) of the total oxylipin pool (Table 4). Thus, an unpaired *t*-test was used to compare free and esterified (i.e., TAG-bound) oxylipin formation / degradation rates to test whether FFAs or TAGs are the preferred substrate for generating oxylipins. As shown in Table 5, the rate (velocity) of esterified LA-derived 9-HODE, 13-HODE and 9-oxo-ODE formation was significantly greater than the rate of free 9-HODE, 13-HODE, and 9-oxo-ODE formation by 1300-fold, 416-fold, and 520-fold, respectively (*p* < 0.05). The rate of the sum of LA-derived oxylipins was 265-fold greater in the esterified pool than the free pool (*p* < 0.05).The velocity of ALA-derived 9-HOTrE and 13-HOTrE was only measurable in the esterified pool, but not the free pool, because these compounds were not detected there.

Turnover was determined by normalizing the velocity of oxylipin formation to the precursor fatty acid substrate concentration within each lipid pool (free or bound). The turnover for total oxylipins was comparable to esterified oxylipins (as expected) so statistical comparisons were only made between the free and esterified pools. As shown in Table 5, the turnover of esterified LA-derived 9-HODE was significantly greater by 28-fold, than the turnover of free 9-HODE (*p* < 0.05). The turnover of other individual LA-metabolites (e.g. 13-HODE, 9-oxo-ODE, etc) and the sum of LA-derived oxylipins was also greater in the esterified pool than the free pool, although these changes were not statistically significant likely due to the high variability between the groups. This suggests that more sampling points are necessary to reduce the variance and accurately estimate LA-derived oxylipin turnover in free and esterified lipid pools. The turnover of esterified ALA-metabolites was measurable and is shown in Table 5; turnover of free ALA-metabolites could not be calculated because they were not detected. Collectively, these data suggest that esterified TAGs are preferentially oxidized over FFAs, favoring the generation of TAG-bound oxylipins over free oxylipins.

## Discussion

In this study, a method for measuring total and free oxylipins in oil was developed and used to establish that during thermal treatment, the esterified fatty acid pool containing TAGs is a better substrate than the FFA pool for generating oxylipins, despite the rapid formation and availability of FFAs due to heat-induced hydrolysis. Our results demonstrate that in heated oil, FFAs produced during thermal treatment are not the major source of oxidized fatty acids (i.e., oxylipins) as previously thought. Rather, TAGs are the major source of oxylipins, and the preferred substrate for lipid oxidation.

With regard to the analytical method, 1 μL of soybean oil was found to be optimal for measuring total and free oxylipins (**Experiments 1 and 3**). Greater oil volumes resulted in lower oxylipin concentrations, likely due to inefficiencies in hydrolysis. Ion suppression/enhancement were minimal and therefore did not account for the observed reductions in oxylipin concentrations at oil volumes greater than 2 µL.

The hydrolysis efficiency was better with NaOH compared to Na_2_CO_3_ (**Experiment 2**). Ion suppression/enhancement and surrogate standard percent recovery were comparable or higher with NaOH than Na_2_CO_3_ (Supplementary Table 3; Supplementary Fig. 2), suggesting that the greater concentrations achieved with NaOH were due to better hydrolysis. This is an agreement with a recent study, which reported that hydrolysis with KOH led to optimal liberation of bound oxylipins in rat plasma^18^.

At baseline (i.e., in non-heated oil), the majority of oxylipins in oil were esterified and approximately 0.2% were present in the free, unbound form (Table 4). Concentrations of LA-derived and ALA-derived oxylipins formed during heating remained significantly higher in the esterified pool compared to the free pool (Table 4). This is in agreement with the greater availability of esterified PUFA precursors for oxidation, compared to free PUFAs. Similar to oxylipins, the majority of fatty acids in oil (>99%) were bound (Table 3), as previously reported^10,11,20^.

Percent FFAs is the current industry standard for measuring lipid oxidation in oil or food samples, because they are presumably more susceptible to auto-, thermal or photo- oxidation than TAGs^21–24^. Although the rate of FFA hydrolysis was high during the 24 h heating period (Fig. 3), oxylipins were generated more rapidly from TAGs than from FFAs (Table 5). To test whether this was due to the greater abundance of esterified PUFA precursors, we normalized the velocity of each oxylipin pool to its free or esterified PUFA precursor substrate. Doing so revealed a greater turnover of several esterified oxylipins than free oxylipins, suggesting that TAGs are preferentially oxidized compared to FFAs, as depicted in Fig. 1 (Pathway 1). These observations are consistent with studies showing that oil TAGs oxidize during heating^25^.

The best fit for the oxylipin and FFA Concentration-Time plots was a linear line (Supplementary Figs. 5 and 6). The observed linearity for most esterified oxylipins is in agreement with studies showing a linear increase in oxidized TAGs during the induction period^26,27^. The goodness of fit (i.e., *R*^2^ value) for free LA and ALA (Supplementary Fig. 5) is consistent with a study which showed that FFAs generated during lipid oxidation followed linear kinetics^28^. These studies, and ours, likely captured the initiation phase of lipid oxidation, which typically follows a linear fit^29^. Longer incubation periods that incorporate the propagation and termination phases are likely to yield non-linear curve fits that allow estimation of maximum reaction velocities using the maximum point of inflection.

Free LA-derived oxylipins were detected during the 24 h heating period, but free ALA-metabolites were not detected, which is why the velocity and turnover of free ALA-metabolites were not determined. Only two ALA-metabolites were screened in this study (9-HOTrE and 13-HOTrE), but others including ALA-epoxides, diols and ketones have been reported to be present in soybean oil^6^. Due to the lack of standard availability at the time, we only determined the formation of 9-HOTrE and 13-HOTrE. Measuring the kinetics of other ALA-metabolites may better inform on the preferential capacity for ALA to oxidize compared to LA.

There are limitations to the kinetic model used in this study. First, it does not capture other reactions shown in Fig. 1, specifically the direct release of TAG-bound oxylipins to generate free oxylipins, or losses in free or bound oxylipins due to the formation of secondary volatile compounds. However, these limitations do not change our conclusion that the apparent turnover of several free oxylipins was negligible or less than the turnover of esterified oxylipins. Future studies using labeled esterified and free PUFA tracers would be necessary to probe tracer dilution into free and bound oxylipins, as well as secondary volatiles, to accurately measure turnover within specific oxidized lipid pools.

In conclusion, our study provides evidence that during heating, TAGs are preferentially oxidized to form oxylipins compared to FFAs, despite the faster hydrolysis and availability of FFAs. This suggests that in oil, the mechanism of lipid oxidation involves TAG utilization as a preferred substrate over FFAs. Therefore, TAG-bound oxylipins may potentially serve as better markers of lipid oxidation in food or oil systems compared to free oxylipins or FFAs.

## Methods

### Soybean oil samples

One bottle of soybean oil (Crisco pure vegetable oil, 1.41 L) was purchased from a local supermarket in Davis. The oil was manufactured by The J.M. Smucker Company (Orrville, USA). The expiry date on the bottle was Feb 10th, 2019. All analysis was done before the expiry date.

### Chemicals and reagents

Methanol (Cat #A454-4), methanol LC-MS grade (Cat #A4564), chloroform (Cat #C607-4), ethyl acetate (Cat #E196-4), acetonitrile (Cat #A9561), hexane (Cat #H303-4), toluene (Cat #T2914), heptane (Cat #H350SK-4), ethyl ether (Cat # 615080010) and 2’,7’-dichlorofluorescein (Cat #AC19153-0050) were obtained from Fisher Scientific (Hampton, NH, USA).

Acetic acid (Cat #695092), HCl (37 M; Cat #320331), butylated hydroxyl toluene (BHT; Cat #W218405-SAMPLE-K), ethylenediaminetetraacetic acid (EDTA; Cat #EDS-100G), triphenyl phosphine (TPP; Cat #3T84409), Na_2_CO_3_ (Cat #791768-500 G), NaOH (Cat #S5881-500G) and cholesterol (Cat #C8667-500MG) were purchased from Sigma-Aldrich (St. Louis., MO, USA).

Deuterated oxylipin standards used for oxylipin quantification were obtained from Cayman chemicals (Ann Arbor, MI, USA). Specifically, d11–11(12)-EpETrE (Cat #10006413), d11-14,15-DiHETrE (Cat #1008040), d4-6-keto-PGF1α (Cat #315210), d4-9-HODE (Cat #338410), d4-LTB4 (Cat #320110), d4-PGE2 (Cat #314010), d4-TXB2 (Cat #319030), d6-20-HETE (Cat #390030), and d8-5-HETE (Cat #334230) were purchased.

The lipid standards, cholesteryl-palmitate (Cat #CH-815), tripalmitin (Cat #T-150), glyceryl triheptadecanoate (Cat #T-155) and palmitic acid (Cat #N-16-A) were purchased from Nu-Chek Prep (Elysian, MN, USA). 1,2-Dipalmitoyl-sn-glycero-3-phosphocholine (Cat #850355C) was obtained from Avanti Polar Lipids (Alabaster, Alabama, USA).

### Study design

Three experiments were carried out to validate the methods for total (free + esterified) and free oxylipin analysis in non-heated oil (**Experiment 1–3**). After method development and validation, the appropriate protocol was used to measure total and free oxylipins in soybean oil samples heated for up to 24 h and collecting aliquots at 0, 1, 4, 8, and 24 h (**Experiment 4**). Total fatty acids and FFAs were also measured in **Experiment 4**. The rate of product formation (velocity) and turnover of FFAs and oxylipins were then calculated.

### Experiment 1

**Experiment 1** was designed to establish the optimal soybean oil volume needed to measure total oxylipins. Previously, total oxylipins were determined by hydrolyzing 10 µL of oil with Na_2_CO_3_ in water: methanol (1:1)^6^. However, no study confirmed whether this volume yielded maximum oxylipin recovery post-hydrolysis. Thus, four different oil volumes—1, 2, 5, and 10 µL—were hydrolyzed in triplicates with Na_2_CO_3_, alongside a blank containing 10 µL chloroform/methanol (2:1 v/v) solvent instead of oil.

Five hundred µL of soybean oil was diluted 10 times in 4.5 mL chloroform/methanol (2:1 v/v). Then, 10, 20, 50, and 100 µL representing 1, 2, 5, and 10 µL of oil were added to 2 mL centrifuge tubes and dried under nitrogen. Ten microliter of antioxidant solution containing 0.2 mg/mL BHT, EDTA, and TPP in water/methanol (1:1 v/v), and 10 µL of surrogate mix standard solution containing 2 µM of d11-11(12)-EpETrE, d11-14,15-DiHETrE, d4-6-keto-PGF1a, d4-9-HODE, d4-LTB4, d4-PGE2, d4-TXB2, d6-20-HETE, and d8-5-HETE in LC-MS grade methanol, were added. The mixture was diluted in 190 µL extraction solvent (0.1% of acetic acid and 0.1% of BHT in methanol) and 200 µL of 0.25 M Na_2_CO_3_ solution in water/methanol (1:1 v/v). The samples were vortexed and heated for 30 min at 60 °C on a heating block. After cooling at room temperature for 5 min, 25 µL acetic acid and 1575 µL MilliQ water were added to the samples, which were vortexed and stored at −20 °C (for ~1 h) until they were extracted with Waters Oasis HLB (3 cc, 60 mg sorbent, 30 µm particle size; Waters Corporation, Milford, CA, USA; Cat #WAT094226) solid phase extraction (SPE) columns. Samples were stored at −20 °C for ~1 h to minimize lipid oxidation, while the SPE columns were washed and preconditioned (next paragraph).

The SPE columns were washed with one column volume of ethyl acetate, followed by two column volumes of methanol, and preconditioned with two column volumes of SPE buffer (0.1% acetic acid, 5% methanol in MilliQ water). The hydrolyzed oil samples were poured onto the columns (one sample per column) and washed with two column volumes of SPE buffer. The columns were dried under vacuum for 20 min. Oxylipins were eluted from the column with 0.5 mL methanol and 1.5 mL ethyl acetate. The oxylipin extract was dried under nitrogen, reconstituted in 100 µL LC-MS grade methanol and filtered by centrifuging at 15,871×*g* (0 °C) in centrifuge tubes containing a filter unit (Ultrafree-MC VV Centrifugal Filter, 0.1 µm; Millipore Sigma, Burlington, MA, USA; Cat #UFC30VV00). The filtered samples were transferred to 2 mL amber LC-MS sample vials (Phenomenex, Torrance, CA, USA; Cat #AR0-3911-13) with pre-slit caps (Phenomenex, Torrance, CA, USA; Cat #AR0-8972-13-B) containing inserts (Waters Corporation, Milford, CA, USA; Cat #WAT094171). Samples were stored at −80 °C until they were analyzed by UPLC-MS/MS as described below.

The percent recovery of each surrogate standard was calculated as follows:

1 $${\mathrm{Percent}}\,{\mathrm{recovery}}\left( {\mathrm{\% }} \right) = \frac{{{\mathrm{Peak}}\,{\mathrm{area}}\,{\mathrm{of}}\,{\mathrm{surrogate}}\,{\mathrm{insample}}}}{{{\mathrm{Peak}}\,{\mathrm{area}}\,{\mathrm{of}}\,{\mathrm{surrogate}}\,{\mathrm{in}}\,{\mathrm{oxylipin}}\,{\mathrm{standard}}}} \times 100$$

Ion suppression/enhancement was determined by making the following 3 solutions: Solution 1 contained 10 µL of oxylipin standard mix (containing the unlabeled analytes and 9 deuterated surrogate standards listed in Supplementary Table 1) and 50 µL of methanol; Solution 2 contained 10 µL of oxylipin standard mix and 50 µL of sample; Solution 3 contained 10 µL of methanol and 50 µL of sample (no standard added). The solutions were run on the UPLC-MS/MS. Ion suppression/enhancement was calculated as follows:

2 $${\mathrm{Ion}}\,{\mathrm{suppression}}/{\mathrm{enhancement}} \, \left( {{\%}}\right) = \frac{{{\mathrm{Peak}}\,{\mathrm{area}}\,{\mathrm{of}}\,{\mathrm{Solution}}\,2}}{{\rm{Peak}}\,{\mathrm{area}}\,{\mathrm{of}}\,{\mathrm{Solution}}\,1 + {\mathrm{Peak}}\,{\mathrm{area}}\,{\mathrm{of}}\,{\mathrm{Solution}}\,3} \times 100$$

### Experiment 2

**Experiment 2** compared the efficiency of hydrolysis with Na_2_CO_3_ to NaOH, which was recently reported to efficiently hydrolyze esterified oxylipins^18,19^. The same concentration of 0.25 M Na_2_CO_3_ and NaOH solution (dissolved in 1:1 methanol/MilliQ water) was used for comparison. One microliter of soybean oil was used for the analysis, based on the findings of **Experiment 1**. Each base condition was done in 5 replicates alongside blank samples composed of 10 µL of 2:1 (v/v) chloroform: methanol (in lieu of oil sample) that were subjected to hydrolysis with each base after drying under nitrogen (*n* = 1 blank per base condition).

Five hundred µL of soybean oil was diluted 10 times in 4.5 mL chloroform/methanol (2:1 v/v). Ten microliter of diluted oil (containing 1 µL of pure oil), 10 µL of antioxidant solution and 10 µL of surrogate standard mix solution were added to 2 mL centrifuge tubes, and the mixture was dried under nitrogen. Then, 190 µL extraction solvent were added to all samples, and 200 µL of 0.25 M Na_2_CO_3_ or NaOH solution in water/methanol (1:1 v/v) were added. The samples were heated for 30 min at 60 °C, and the hydrolyzed oxylipins were cooled, acidified with 25 µL acetic acid and 1575 µL MilliQ water extracted, subjected to SPE on the Oasis HLB columns, and analyzed by UPLC-MS/MS. Percent recovery and ion suppression were determined as described above.

### Experiment 3

**Experiment 3** tested the appropriate soybean oil volume needed to separate free from bound oxylipins. Waters Silica columns (100 mg; Waters, Milford, MA; Cat #WAT023595) were used, because they trap polar lipids (oxylipins) and elute non-polar lipids (e.g., TAGs). Four oil volumes (1, 2, 5, and 8 µL) were subjected to silica SPE separation in triplicates as described below. A blank containing 80 µL of chloroform was treated the same way.

The oils were prepared by diluting soybean oil (100 µL) 10 times in 900 µL chloroform. Then, 10, 20, 50, and 80 µL of diluted oil representing 1, 2, 5, and 8 µL of oil were added to 2 mL centrifuge tubes containing 10 µL of antioxidant solution and 10 µL of surrogate standards mix solution. The samples were vortexed and loaded onto Silica SPE columns pre-washed with two column volumes of methanol, and pre-conditioned with two column volumes of chloroform. The columns were rinsed with 1.5 mL chloroform to elute neutral lipids containing TAGs and sterol esters. Free oxylipins were eluted with 1.5 mL methanol. The extracted free oxylipins were dried under nitrogen and reconstituted in 100 µL LC-MS grade methanol and analyzed by UPLC-MS/MS, as described above.

To confirm the absence of free oxylipins in neutral lipids, the chloroform fraction was also dried under nitrogen, reconstituted, filtered and injected directly into the UPLC-MS/MS.

The percent recoveries of surrogate standards and ion suppression/enhancement were determined in the free oxylipin extract as described in **Experiment 1**.

### Experiment 4

**Experiment 4** utilized optimal conditions from Experiments 1 to 3 to determine total and free oxylipin concentrations in soybean oil heated for 24 h (5 replicates per time-point). Total fatty acids and FFAs in heated soybean oil were also measured (see below). Based on **Experiments 1 to 3**, 1 µL of soybean oil was used to separate free from esterified oxylipins, and 0.25 M NaOH solution was used to hydrolyze bound oxylipin.

Five replicates of 5.1 mL soybean oil were heated at 100 °C for 24 h in 8 mL amber glass tubes (17 mm diameter, 63 mm height; Fisher Scientific, Hampton, NH, USA; Cat# 14-955-332). One hundred microliter of oil was taken from each tube at 0 h (room temperature) as baseline, and stored in −80 °C. The vials were then heated with no caps on them, on a heating block maintained at 100 °C for 24 h. At 1, 4, 8, and 24 h of heating, 100 µL of soybean oil were aliquoted into to 2 mL amber vials with non-slit caps (Phenomenex, Torrance, CA, USA; Cat #AR0-8951-13-B) containing inserts, cooled at room temperature for 10 min and stored at −80 °C. The rationale for sampling at 1, 4, 8, and 24 h is based on a preliminary study from our group showing that heating at 100 °C increases the production of oxylipins by 4 h^30^. The oil temperature was measured during the heating process using an additional 6th tube containing 5.1 mL of soybean oil; 100 µL of oil was removed from the tube at each sampling point, similar to the tubes sampled for oxylipin analysis.

Ten microliter of the heated oils were diluted 10 times in 90 µL chloroform/methanol (2:1). Total oxylipins were determined by hydrolyzing 10 µL of the diluted oil (containing 1 µL of soybean oil) in 0.25 M NaOH solution as described in **Experiment 2**. For free oxylipin analysis, 10 µL of the heated oils were diluted 10 times in 90 µL chloroform, and 10 µL of the diluted oil (1 µL of soybean oil) was analyzed as described in **Experiment 3**. Surrogate standard percent recovery and ion suppression/enhancement were determined.

Total fatty acid and FFAs in the heated oil were analyzed as described below.

### UPLC-MS/MS analysis

Oxylipins were analyzed on an Agilent 1290 Infinity (Agilent Technologies, Santa Clara, CA, USA) UPLC system coupled to an Agilent 6460 Triple Quadropole mass-spectrometer (MS; Agilent Technologies, Santa Clara, CA, USA). The UPLC was equipped with an Agilent ZORBAX Eclipse Plus C18 column (2.1 × 150 mm, 1.8 μm particle size; Agilent Technologies, Santa Clara, CA, USA; Cat #959759-902). The triple quadrupole was operated in negative electrospray ionization mode and using optimized dynamic Multiple Reaction Monitoring (dMRM) conditions (Supplementary Table 1). The temperature of the auto-sampler and the column was kept at 4 °C and 45 °C, respectively. MilliQ water containing 0.1% acetic acid was used as mobile phase A, while acetonitrile/methanol (80/15 v/v) with 0.1% acetic acid was used as mobile phase B. Mobile phase B was held at 35% initially, and increased to 40% at 3 min at a flow rate of 0.3 mL/min. It was increased to 48% at 4 min, 60% at 10 min, 70% at 20 min, and 85% at 24 min, and held at 85% until 24.5 min. The flow rate during this period was 0.25 mL/min. Mobile phase B was increased to 100% from 24.6 min to 26 min, and decreased to 35% at 26.1 min, at 0.35 mL/min. The total run time was 28 min.

### Total fatty acid determination

The oil samples from **Experiment 4** were subjected to fatty acid analysis. Ten µL of oil was dissolved in 90 µL of chloroform/methanol (2:1 v/v). Twenty microliter of the diluted oil (amounting to 2 µL pure oil) was added to 8 mL glass tubes (100 mm diameter, 13 mm height; Thomas Scientific, Swedesboro, NJ, USA; Cat #9215D32) containing glyceryl triheptadecanoate as an internal standard. The standard was derived from a stock mix containing 10.2 mg/mL in chloroform/methanol (2:1 v/v), from which 20 µL were added to each sample (amounting to 0.204 mg standard per sample). The mixtures were vortexed and dried under nitrogen. Then, 400 µL of toluene, 3 mL of methanol and 600 µL of concentrated HCl (37%) in methanol (8:92 v/v) were added. The samples were vortexed, capped and transesterified by heating for 60 min at 90 °C on a heating block. The samples were then cooled at room temperature for 5 min. One mililiter of hexane and 1 mL of MilliQ water were added. Samples were vortexed and the layers allowed to separate for 10 min at room temperature. Nine hundred microliter of the hexane upper layer were transferred to 1.5 mL Eppendorf tubes containing 450 µL of MilliQ water. The mixture was vortexed and centrifuged for 2 min at 15,871 × *g* (5424 R Centrifuge; Eppendorf AG, Hamburg, Germany). The upper hexane upper layer (~800 µL) was transferred to new 1.5 mL Eppendorf tubes, dried under nitrogen, reconstituted in 1 mL of hexane and transferred to 2 mL amber vials with non-slit caps. Samples were stored at −20 °C for up to 1 week prior to analysis by gas chromatography coupled to flame ionization detection (GC-FID).

### FFA determination

FFAs were separated using thin layer chromatography (TLC). Oil samples (10 µL) were diluted in 90 µL chloroform/methanol (2:1 v/v), and 30 µL (containing 3 µL oil) were plated on TLC silica gel glass plates (EMD Milipore, Billerica, MA, USA; Cat #M1057210001) pre-washed with 150 mL of chloroform/methanol (2:1 v/v) and heated overnight at 80 °C under vacuum to remove moisture. The 3 µL oil volume was based on a preliminary experiment which showed that this volume yielded visually notable separation of FFAs from other lipids (Supplementary Fig. 7). Oil samples were plated alongside 10 µL TLC standard mix solution containing 10 mg/mL of cholesteryl-palmitate, tripalmitin, palmitic acid, cholesterol, and 1,2-dipalmitoyl-sn-glycero-3-phosphocholine dissolved in chloroform/methanol (2:1 v/v), to identify the bands. The bands were resolved in TLC tanks using 103 mL of heptane/ethyl ether/acetic acid (60:40:3, v/v/v) solvent mixture. Two plates were placed per tank and each plate was plated with 3 oil samples (~4 cm wide) and 1 blank consisting of 30 µL of chloroform/methanol (2:1 v/v) solvent.

Samples were loaded onto the TLC plates in a randomized manner, determined using Microsoft Excel 2010 Random Formula. The solvent was allowed to migrate to approximately 1–2 cm below the top edge of the plate. The plates were dried for a few minutes under a fume hood and sprayed with 0.02% 2’,7’-dichlorofluorescein solution in methanol. The different lipid bands were revealed under ultraviolet light. The FFA bands were circled with a pencil, scraped and transferred to 8 mL glass tubes containing 0.00133 mg of free heptadecanoic acid as an internal standard (obtained by spiking each test-tube with 100 µL of 0.0133 mg/mL standard in 2:1 v/v chloroform/methanol). Toluene (400 µL) was added and the samples transesterified as described above. The samples were reconstituted in 100 µL of hexane and transferred to 2 mL amber vials containing inserts with non-slit caps. Samples were stored at −20 °C until GC-FID analysis.

### Fatty acid analysis with gas chromatography

Fatty acid methyl esters were analyzed on a Perkin Elmer Clarus 500 GC-FID system (Perkin Elmer, Shelton, CT, USA) equipped with a DB-FFAP polyethylene glycol fused capillary column (30 m × 0.25 mm inner diameter × 0.25 μm film thickness; Agilent Technologies, Santa Clara, CA, USA). The injection volume was 1 μL and 2 μL per sample for total fatty acids and FFAs, respectively. Helium was used as the carrier gas with the flow rate set at 1.3 mL/min. The temperature of the injector was maintained at 240 °C, and the detector was maintained at 300 °C. The column temperature was programmed as follows: initial hold at 80 °C for 2 min, increase from 80 to 185 °C at 10 °C/min, increase to 240 °C at 5 °C/min, and hold at 240 °C for 13 min. A mix of 31 fatty acid methyl ester standards (Nu-Chek Prep, Elysian, MN, USA) was run under the same conditions to identify fatty acids based on retention time. The retention times of the main fatty acids observed in soybean oil are in Supplementary Table 6.

### Kinetic calculations

Esterified oxylipin (Eq. 3) and fatty acid concentrations (Eq. 4) were derived from the difference between total and free oxylipins or fatty as follows:

3 $$C_{{\mathrm{Esterified}}\,{\mathrm{oxylipin}}} = C_{{\mathrm{Total}}\,{\mathrm{oxylipin}}} - C_{{\mathrm{Free}}\,{\mathrm{oxylipin}}}$$

4 $$C_{{\mathrm{Esterified}}\,{\mathrm{fatty}}\,{\mathrm{acid}}} = C_{{\mathrm{Total}}\,{\mathrm{fatty}}\,{\mathrm{acid}}} - C_{{\mathrm{Free}}\,{\mathrm{fatty}}\,{\mathrm{acid}}}$$

where *C is* concentration of an oxylipin or a fatty acid in pmol·μL^−1^ or nmol·μL^−1^, respectively.

A linear model was found to best fit the oxylipins and FFAs produced during the heating process. The velocity (*v*) or reaction rate^31^, defined as the amount of oxylipin or FFA formed per unit time, was calculated by Eq. 5.

5 $$v = \frac{{dC}}{{dt}} = {\mathrm{slope}}$$

where *v* is the velocity (rate) of oxylipin or FFA formation in pmol·μL^−1^·min^−1^, *C* is the oxylipin or FFA concentration in pmol·μL^−1^, *t* is the heating time in min, and the slope is in pmol·μL^−1^·min^−1^, as obtained from the linear fit.

Turnover (*F*), defined as the rate of oxylipin formed per unit substrate (i.e., free or esterified LA or ALA precursors), was calculated using Eq. 6 or Eq. 7.

6 $$F_{{\mathrm{LA}}\,{\mathrm{derived}}\,{\mathrm{oxylipin}}} = \frac{{v_{{\mathrm{LA}} - {\mathrm{derived}}\,{\mathrm{oxylipin}}}}}{{C_{{\mathrm{LA}}}}}$$

7 $$F_{{\mathrm{ALA}}\,{\mathrm{derived}}\,{\mathrm{oxylipin}}} = \frac{{v_{{\mathrm{ALA}} - {\mathrm{derived}}\,{\mathrm{oxylipin}}}}}{{C_{{\mathrm{ALA}}}}}$$

where *F* is turnover in min^−1^, *v* is the velocity of total, free or esterified oxylipin formation in pmol·μL^−1^·min^−1^, and *C* is the concentration of the precursor total, free or esterified LA or ALA substrate in nmol·μL^−1^, at 0 h.

The turnover of FFA, defined as the rate of FFA formed per esterified precursor TAG substrate, was calculated using Eq. 8.

8 $$F_{{\mathrm{FFA}}} = \frac{{v_{{\mathrm{FFA}}}}}{{C_{{\mathrm{esterified}}\,{\mathrm{fatty}}\,{\mathrm{acid}}}}}$$

where *F* is the turnover in min^−1^, *v* is the velocity of FFA formation in pmol·μL^−1^·min^−1^, and *C* is the concentration of the esterified LA or ALA substrate in nmol·μL^−1^, at 0 h.

### Data and statistical analysis

All data are expressed as mean ± standard deviation (SD). Data were analyzed on Graph Pad Prism 7.04 (La Jolla, CA, USA) or Microsoft Excel 2010 (Microsoft Cooperation, Seattle, WA, USA). In Experiment 3, there were missing data for a few compounds in 1 out of 3 samples per group, so they were imputed by dividing the limits of quantification (LOQ) by the square root of 2. The LOQ is the lowest observable concentration on the standard curve.

For **Experiment 1** and **Experiment 3**, thelowest observable concentration on effects of different soybean oil volumes on total and free oxylipin concentrations were determined by one-way ANOVA followed by Tukey’s post-hoc test. For **Experiment 2**, the effect of the two types of bases used for hydrolysis on total oxylipin concentrations was compared by unpaired *t*-test. In **Experiment 4**, a one-way ANOVA followed by Tukey’s post-hoc test was used to compare the rate of hydrolysis and turnover of FFAs. A repeated measures one-way ANOVA followed by Tukey’s post-hoc test was used to determine the effect of heat on total and free oxylipin and fatty acid concentrations over time. An unpaired *t*-test was applied to compare the velocity and turnover of esterified versus free oxylipins.

Statistical significance was set at *p* < 0.05.

### Reporting summary

Further information on research design is available in the Nature Research Reporting Summary linked to this article.

## Supplementary information

Reporting summary

Supplementary Material

## Data Availability

All data generated or analyzed during this study are included in this article or provided in the supplementary files. Additional raw data are available upon request.
