# Publisher Correction: Triacylglycerols are preferentially oxidized over free fatty acids in heated soybean oil

**DOI:** 10.1038/s41538-021-00095-2

**Published:** 2021-04-28

**Authors:** Qing Shen, Zhichao Zhang, Shiva Emami, Jianchu Chen, Juliana Maria Leite Nobrega de Moura Bell, Ameer Y. Taha

**Affiliations:** 1 Department of Food Science and Technology, College of Agriculture and Environmental Sciences, University of California Davis, Davis, CA USA; 2 College of Biosystems Engineering and Food Science, Zhejiang University, Hangzhou, China; 3 Department of Biological and Agricultural Engineering, University of California Davis, Davis, CA USA

**Keywords:** Chemistry, Chemical biology

Correction to: *npj Science of Food* 10.1038/s41538-021-00086-3, published online 1 April 2021

The original version of the published Article contained an error in the abstract text. This has been corrected in the HTML and PDF version of the Article.

