# Supplementary material for: Triacylglycerols are preferentially oxidized over free fatty acids in heated soybean oil
Source: NPJ Sci Food. 2021 Apr 1;5:7. doi: 10.1038/s41538-021-00086-3 (PMC8016982; doi:10.1038/s41538-021-00086-3)
Supplement: Supplementary file 1 — Reporting summary [file 41538_2021_86_MOESM1_ESM.pdf]

nature research

Corresponding author(s): Dr Ameer Taha

Last updated by author(s): Nov 2, 2020

Reporting Summary

Nature Research wishes to improve the reproducibility of the work that we publish. This form provides structure for consistency and transparency in reporting. For further information on Nature Research policies, see our Editorial Policies and the Editorial Policy Checklist.

Statistics

For all statistical analyses, confirm that the following items are present in the figure legend, table legend, main text, or Methods section.

n/a	Confirmed	
☐	☒	The exact sample size (n) for each experimental group/condition, given as a discrete number and unit of measurement
☐	☒	A statement on whether measurements were taken from distinct samples or whether the same sample was measured repeatedly
☐	☒	The statistical test(s) used AND whether they are one- or two-sided<br>*Only common tests should be described solely by name; describe more complex techniques in the Methods section.*
☒	☐	A description of all covariates tested
☒	☐	A description of any assumptions or corrections, such as tests of normality and adjustment for multiple comparisons
☐	☒	A full description of the statistical parameters including central tendency (e.g. means) or other basic estimates (e.g. regression coefficient) AND variation (e.g. standard deviation) or associated estimates of uncertainty (e.g. confidence intervals)
☐	☒	For null hypothesis testing, the test statistic (e.g. F, t, r) with confidence intervals, effect sizes, degrees of freedom and P value noted<br>*Give P values as exact values whenever suitable.*
☒	☐	For Bayesian analysis, information on the choice of priors and Markov chain Monte Carlo settings
☒	☐	For hierarchical and complex designs, identification of the appropriate level for tests and full reporting of outcomes
☒	☐	Estimates of effect sizes (e.g. Cohen's d, Pearson's r), indicating how they were calculated

Our web collection on statistics for biologists contains articles on many of the points above.

Software and code

Policy information about availability of computer code

Data collection	Data were collected by Agilent MassHunter Workstation Software (Agilent Technologies, Santa Clara, CA, USA).
Data analysis	Data were analyzed on Graph Pad Prism 7.04 (La Jolla, CA, USA) or Microsoft Excel 2010 (Microsoft Cooperation, Seattle, WA, USA).

For manuscripts utilizing custom algorithms or software that are central to the research but not yet described in published literature, software must be made available to editors and reviewers. We strongly encourage code deposition in a community repository (e.g. GitHub). See the Nature Research guidelines for submitting code & software for further information.

Data

Policy information about availability of data

All manuscripts must include a data availability statement. This statement should provide the following information, where applicable:

- Accession codes, unique identifiers, or web links for publicly available datasets
- A list of figures that have associated raw data
- A description of any restrictions on data availability

All data generated or analyzed during this study are included in this article or provided in the supplementary files. Additional raw data are available upon request.

Field-specific reporting

Ecological, evolutionary & environmental sciences study design

All studies must disclose on these points even when the disclosure is negative.

Study description	This study validated methods for free and esterified oxylipin analysis in oils, and used the appropriate method to determine the kinetics of oxidation within free and esterified lipid fractions in soybean oil. Three experiments were carried out to validate the hydrolysis a and analytical methods used (Experiment 1-3). Experiment 1 was designed to establish the optimal soybean oil volume (1,2,5 or 10 μL) needed to measure total (free + esterified) oxylipins following hydrolysis with sodium carbonate (n=3). Experiment 2 compared the hydrolysis efficiency of two bases (sodium carbonate versus sodium hydroxide; n=3 per base). Experiment 3 tested the appropriate soybean oil volume (1,2,5 or 8 μL) needed to separate free from esterified oxylipins (n=3). Following method validation, the appropriate protocol was used to measure total and free oxylipins in soybean oil samples heated for 24 h (Experiment 4). Aliquots were collected at 0, 1, 4, 8 and 24 h (n=5 per time-point), and free and total oxylipins and fatty acids were measured. Kinetic analysis was used to calculate rate of product formation (velocity) and turnover. Esterified fatty acids minimally changed over time. Free fatty acids, which are precursors to free oxylipins, were generated more rapidly than free and esterified oxylipins. However, the rate and turnover of esterified oxylipin formation was greater than that of free oxylipins. The data suggest that esterified lipids (i.e. triacylglycerols) oxidize faster than free fatty acids.
Research sample	Soybean oil (Crisco pure vegetable oil, 1.41 L) manufactured by The J.M. Smucker Company (Orrville, USA).
Sampling strategy	We used a sample size comparable to a previous study that performed similar measurements (PMID: 28157307). However, no formal sample-size calculation was performed.
Data collection	Data were collected by Agilent MassHunter Workstation Software (Agilent Technologies, Santa Clara, CA, USA).
Timing and spatial scale	Data for Experiment 1 were collected from Jan 31st to Feb 1st, 2019; data for Experiment 2 were collected from March 6th to 9th,2019; data for Experiment 3 were collected from April 26th to 28th,2019; data for Experiment 4 were collected from April 6th to July 4th,2019.
Data exclusions	No data were excluded from the analysis.
Reproducibility	In Table 1, we compared results with our previous study (PMID: 28157307) and showed that our values were reproducible. Additionally, within this study, our values were cosistent between experiments.
Randomization	Samples were randomized by using Microsoft Excel 2010 Random Formula.
Blinding	Samples were labeled with a randomized sequence during data acquisition and analysis.

Did the study involve field work? ☐ Yes ☒ No

Reporting for specific materials, systems and methods

We require information from authors about some types of materials, experimental systems and methods used in many studies. Here, indicate whether each material, system or method listed is relevant to your study. If you are not sure if a list item applies to your research, read the appropriate section before selecting a response.

Materials & experimental systems

n/a	Involved in the study	
☒	☐	Antibodies
☒	☐	Eukaryotic cell lines
☒	☐	Palaeontology and archaeology
☒	☐	Animals and other organisms
☒	☐	Human research participants
☒	☐	Clinical data
☒	☐	Dual use research of concern

Methods

n/a	Involved in the study	
☒	☐	ChIP-seq
☒	☐	Flow cytometry
☒	☐	MRI-based neuroimaging
