# Supplementary material for: Triacylglycerols are preferentially oxidized over free fatty acids in heated soybean oil
Source: NPJ Sci Food. 2021 Apr 1;5:7. doi: 10.1038/s41538-021-00086-3 (PMC8016982; doi:10.1038/s41538-021-00086-3)
Supplement: Supplementary file 2 — Supplementary Material [file 41538_2021_86_MOESM2_ESM.pdf]

Supplementary information

Triacylglycerols are preferentially oxidized over free fatty acids in heated soybean oil

Qing Shen[a,b], Zhichao Zhang[a], Shiva Emami[a], Jianchu Chen[b], Juliana Maria Leite Nobrega de Moura Bell[a,c] and Ameer Y. Taha[a,]*

[a] Department of Food Science and Technology, College of Agriculture and Environmental Sciences, University of California, Davis 95616, CA, United States

[b] College of Biosystems Engineering and Food Science, Zhejiang University, Hangzhou 310058, China

[c] Department of Biological and Agricultural Engineering, University of California, Davis, One Shields Avenue, Davis, CA 95616, USA

*Corresponding author:

Dr. Ameer Y. Taha

Department of Food Science and Technology, College of Agriculture and Environmental Sciences, University of California Davis, Davis 95616, CA, United States

Phone: (+1) 530-752-7096

Email: ataha@ucdavis.edu

Supplementary Table 1. Retention time, parent ion, product ion, fragmentor voltage and collision energy of the deuterated surrogate standards and unlabeled oxylipins.

Name	Surrogate used	RT (min)	Parent ion (m/z)	Product ion (m/z)	Fragmentor voltage (V)	Collision energy (V)
**Surrogate**						
d-11-11(12)-EpEtrE	NA	23.3	330.2	207.1	130	7
d11-14,15-DiHETrE	NA	13.3	348.2	207.1	110	12
d4-6-keto-PGF1a	NA	4.3	373.3	157.1	110	25
d4-9-HODE	NA	17.5	299.2	172.3	80	10
d4-LTB4	NA	11.5	339.2	197.2	80	10
d4-PGE2	NA	6.7	355.2	275.3	90	10
d4-TXB2	NA	5.8	373.3	173.2	100	12
d6-20-HETE	NA	15.9	325.2	281.2	70	12
d8-5-HETE	NA	20.6	327.2	116.1	70	7
**LA-derived oxylipins**						
9-HODE	d4-9-HODE	17.5	295.2	171.1	110	12
13-HODE	d4-9-HODE	17.5	295.2	195.2	100	7
9-oxo-ODE	d6-20-HETE	19.4	293.2	185.1	100	12
13-oxo-ODE	d6-20-HETE	18.6	293.2	195.1	120	15
9(10)-EpOME	d-11-11(12)-EpEtrE	22.2	295.3	171.1	100	12
12(13)-EpOME	d-11-11(12)-EpEtrE	21.8	295.3	195.2	100	12
9,10-DiHOME	d11-14,15-DiHETrE	12.7	313.2	201.2	120	12
12,13-DiHOME	d11-14,15-DiHETrE	12.2	313.2	183.2	110	15
9,10,13-TriHOME	d4-PGE2	6.4	329.2	171.1	120	8
9,12,13-TriHOME	d4-PGE2	6.3	329.2	211.1	120	8
**ALA-derived oxylipins**						
9-HOTrE	d4-9-HODE	14.7	293.2	171.2	120	10
13-HOTrE	d4-9-HODE	15.1	293.2	195.1	100	10

Supplementary Table 2. Matrix effects (i.e. % ion suppression or enhancement) following oxylipin analysis in different soybean oil volumes. Ion enhancement: >100%; ion suppression: <100%. Data are n=1 per group.

	Volume of soybean oil (µL)			
	**1**	**2**	**5**	**10**
**Total LA-derived oxylipins**				
9-HODE	114	124	134	116
13-HODE	125	143	146	133
9-oxo-ODE	108	113	105	103
13-oxo-ODE	115	120	133	136
9(10)-EpOME	116	151	131	129
12(13)-EpOME	112	136	119	118
9,10-DiHOME	109	127	117	115
12,13-DiHOME	88	95	100	99
9,10,13-TriHOME	99	103	111	101
9,12,13-TriHOME	103	108	122	104
**Total ALA-derived oxylipins**				
9-HOTrE	105	121	129	115
13-HOTrE	111	122	136	119

Supplementary Table 3. Matrix effects (i.e. % ion suppression or enhancement) following hydrolysis of soybean oil with different base types. Ion suppression data are expressed at % of control. Ion enhancement: >100%; ion suppression: <100%. Data are n=1 per base condition.

**% of control**	**Base hydrolysis type**	
	**Na_2CO_3**	**NaOH**
**Total LA-derived oxylipin**		
9-HODE	104	127
13-HODE	110	132
9-oxo-ODE	99	101
13-oxo-ODE	126	102
9(10)-EpOME	100	102
12(13)-EpOME	98	104
9,10-DiHOME	104	113
12,13-DiHOME	103	110
9,10,13-TriHOME	101	99
9,12,13-TriHOME	105	106
**Total ALA-derived oxylipin**		
9-HOTrE	101	120
13-HOTrE	113	127

Supplementary Table 4. Matrix effects (i.e. % ion suppression or enhancement) following separation of free oxylipins from esterified oxylipins in 1, 2, 5 or 8 µL soybean oil with silica columns. Data are n=1 per oil volume. Ion enhancement: >100%; ion suppression: <100%.

% of control	Volume of soybean oil (µL)			
	1	2	5	8
**Free LA-derived oxylipin**				
9-HODE	96	102	107	110
13-HODE	99	105	110	113
9-oxo-ODE	100	105	101	109
13-oxo-ODE	92	97	106	103
9(10)-EpOME	92	100	98	102
12(13)-EpOME	86	96	99	99
9,10-DiHOME	96	106	109	112
12,13-DiHOME	78	81	91	90
9,10,13-TriHOME	99	107	111	116
9,12,13-TriHOME	99	109	110	117
**Free ALA-derived oxylipin**				
9-HOTrE	104	113	115	118
13-HOTrE	100	107	111	114

Supplementary Table 5. Raw UPLC-MS/MS chromatograms of each of the 9 deuterated surrogate standards recovered in the polar and neutral lipid fractions following silica column separation of 1, 2, 5, and 8 µL of soybean oil.

Surrogate	Oil volume (µL)	Polar lipid fraction	Neutral lipid fraction
d11-11(12)-EpETrE	1	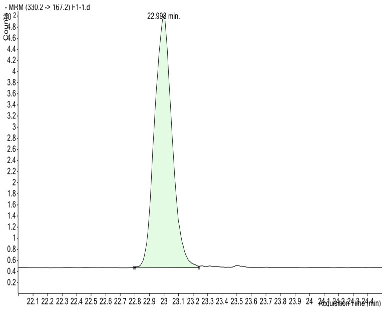	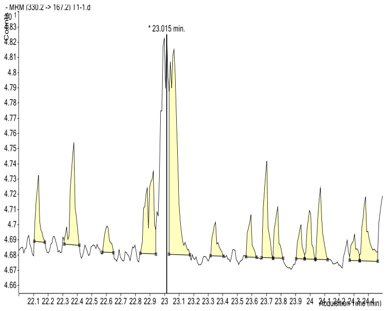
	2	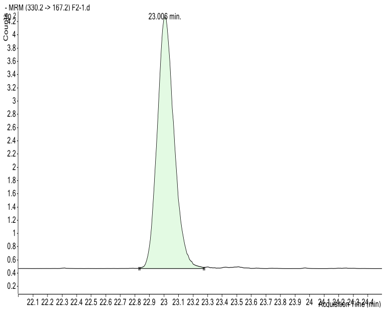	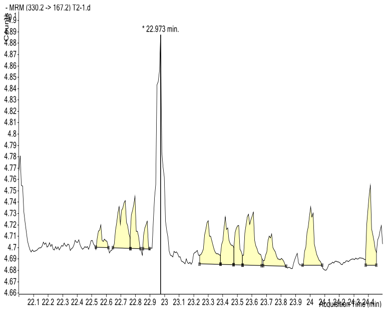
	5	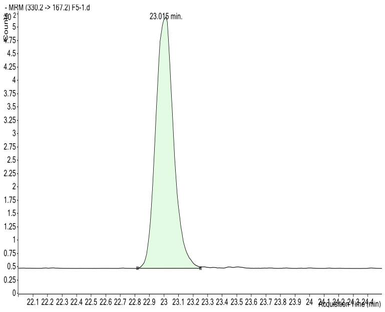	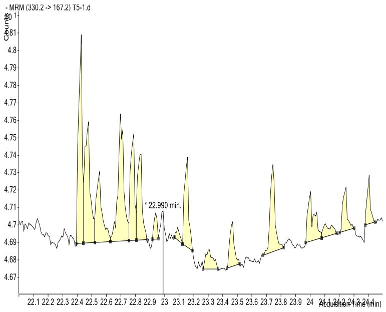
	8	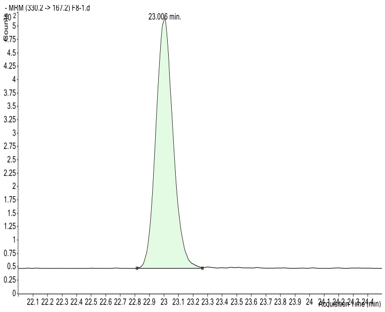	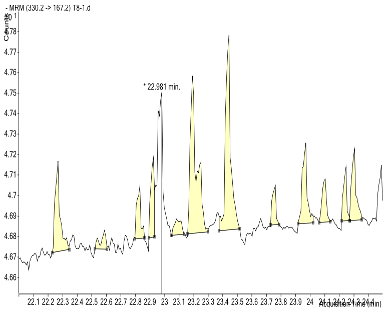

Surrogate	Oil volume (μL)	Polar lipid fraction	Neutral lipid fraction
d11-14,15-DiHETrE	1	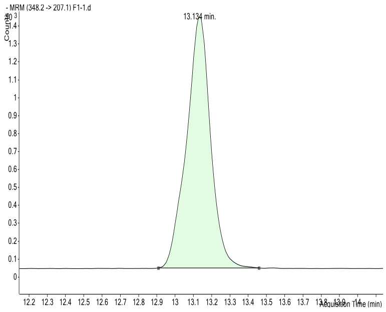	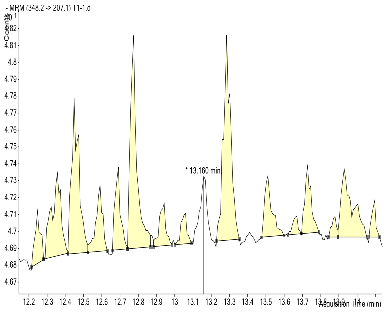
	2	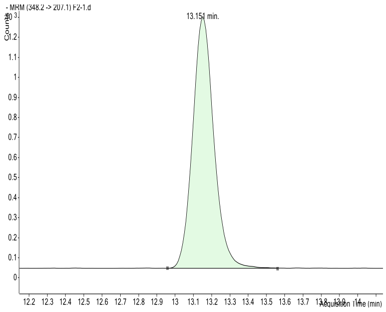	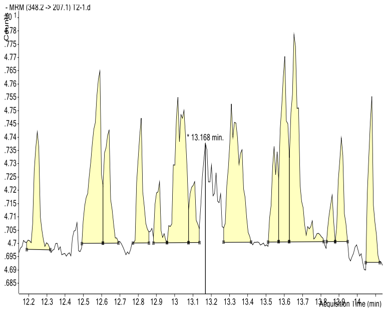
	5	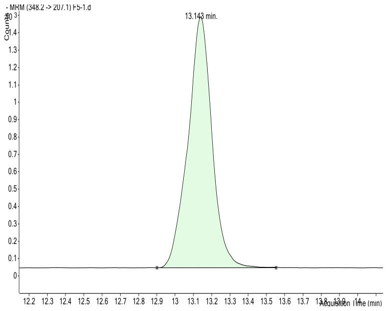	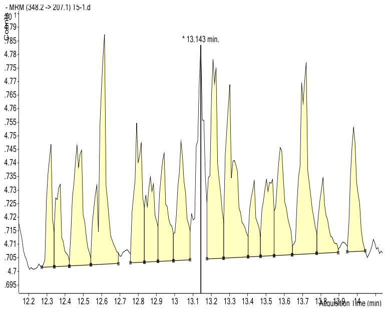
	8	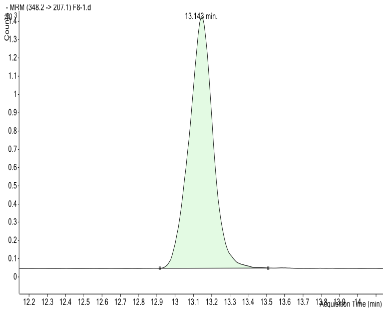	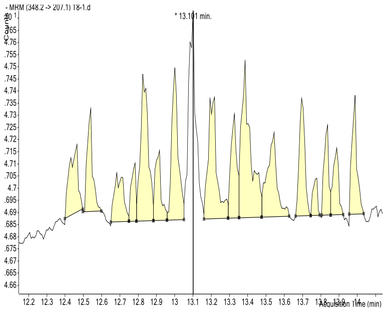
d4-6-keto-PGF1a	1	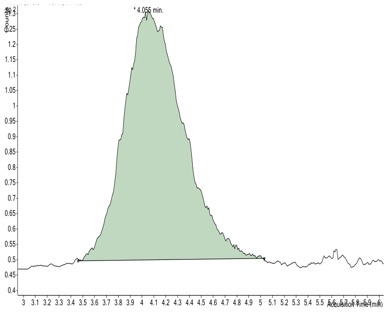	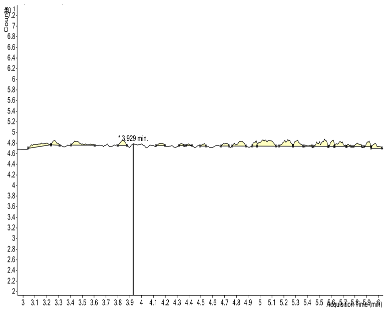

Surrogate	Oil volume (μL)	Polar lipid fraction	Neutral lipid fraction
	2	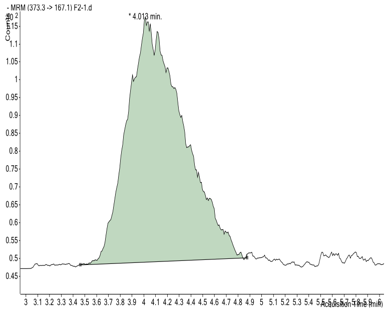	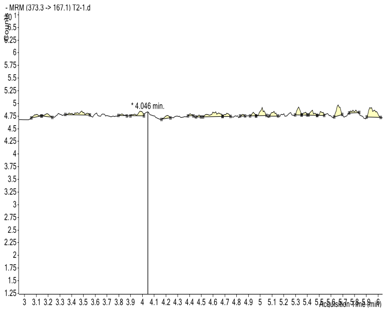
	5	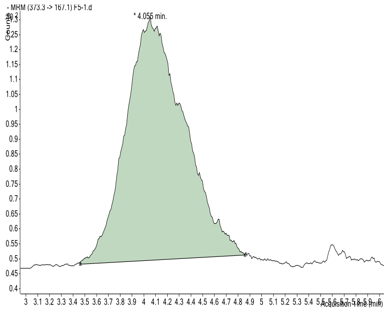	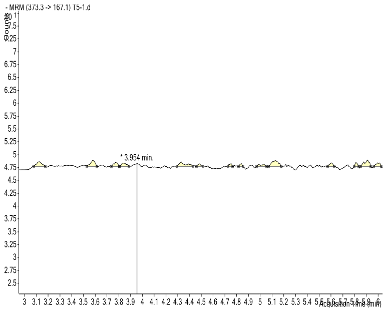
	8	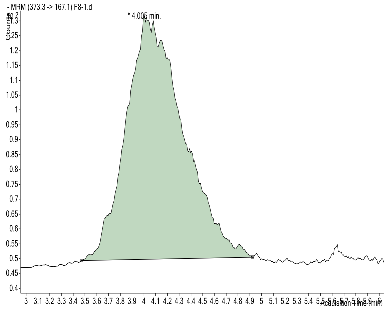	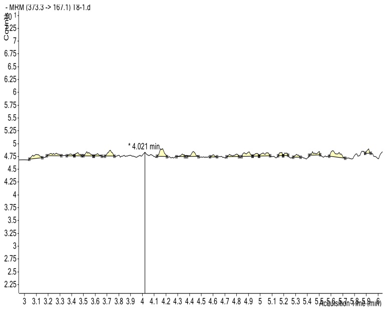
d4-9-HODE	1	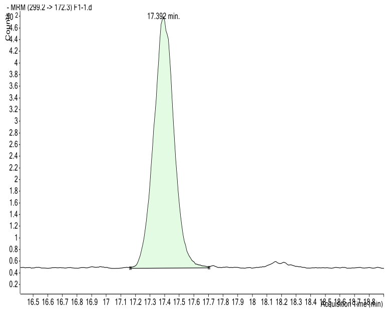	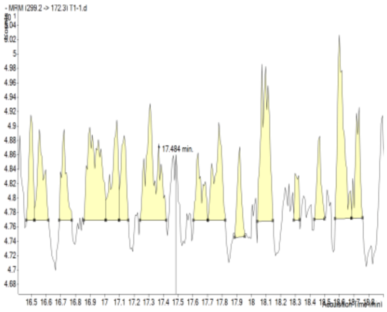
	2	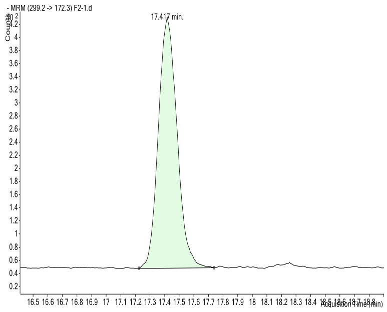	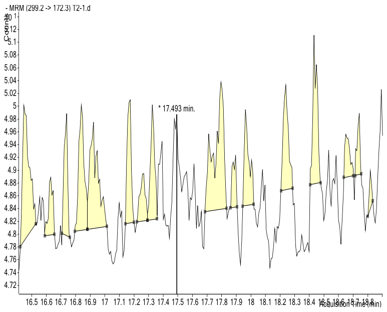

Surrogate	Oil volume (µL)	Polar lipid fraction	Neutral lipid fraction
	5	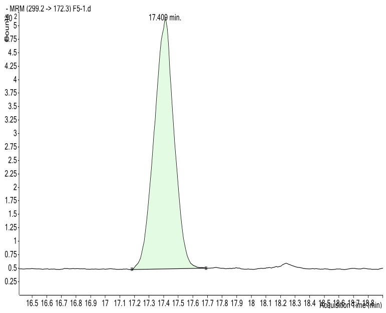	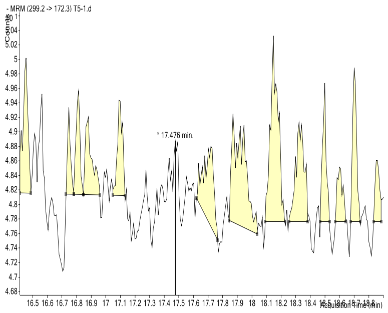
	8	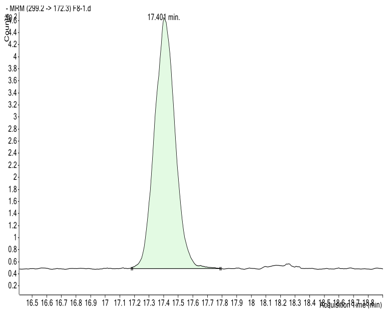	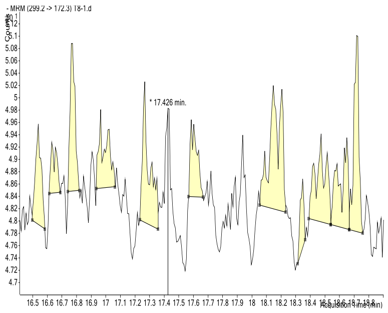
d4-LTB4	1	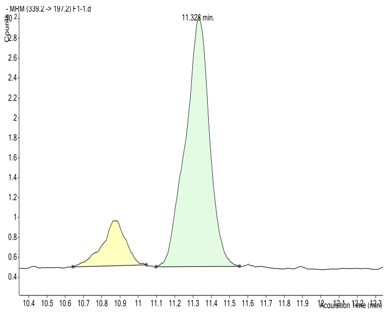	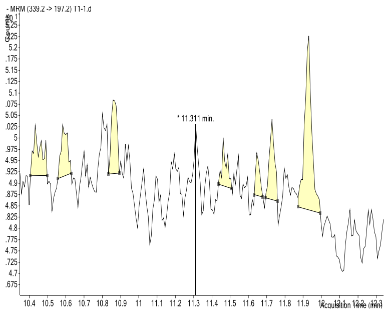
	2	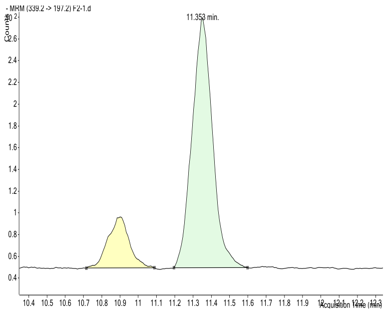	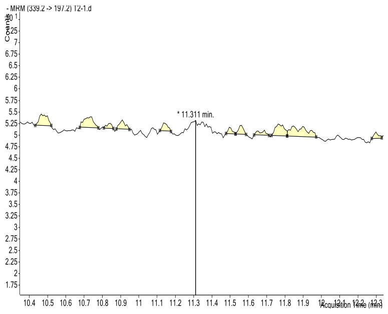
	5	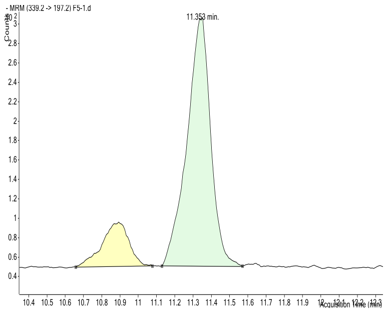	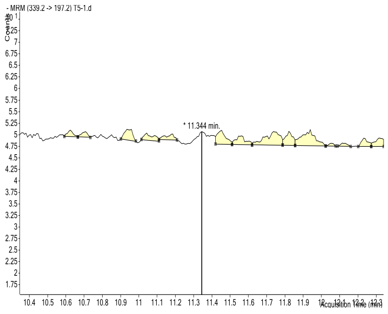

Surrogate	Oil volume (μL)	Polar lipid fraction	Neutral lipid fraction
	8	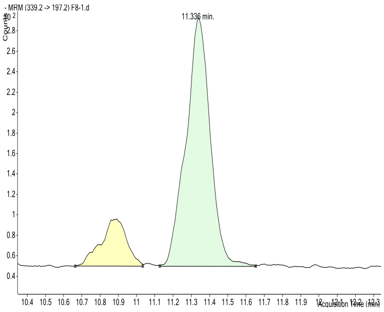	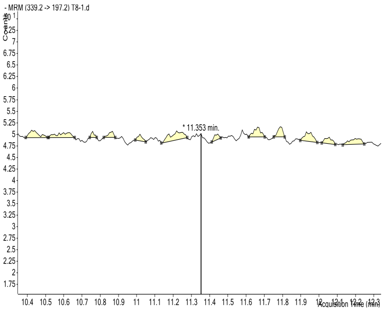
d4-PGE2	1	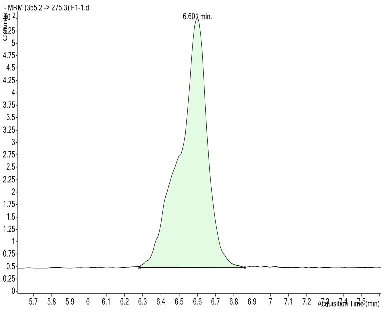	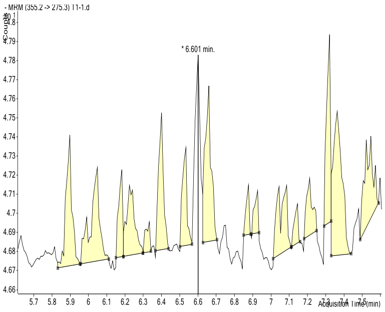
	2	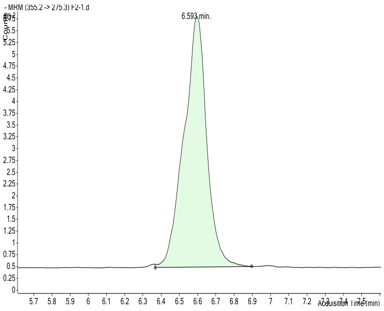	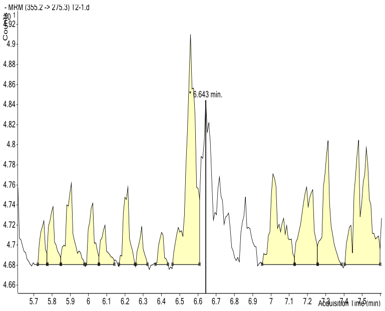
	5	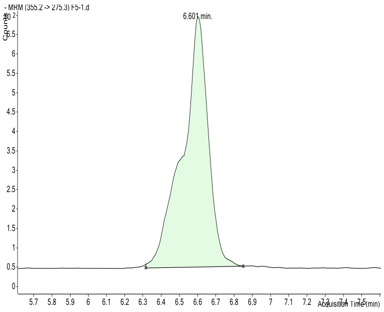	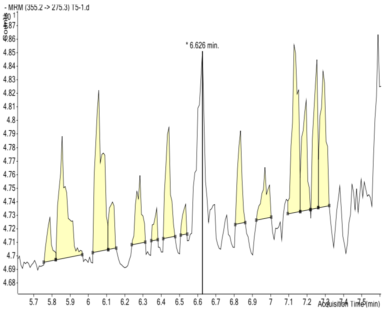
	8	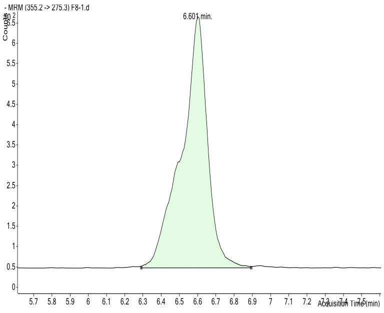	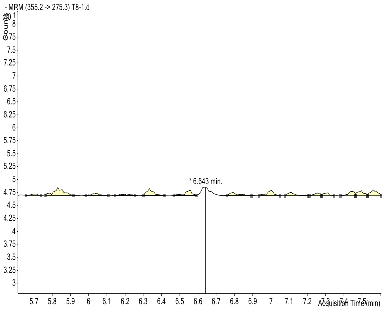

Surrogate	Oil volume (µL)	Polar lipid fraction	Neutral lipid fraction
d4-TXB2	1	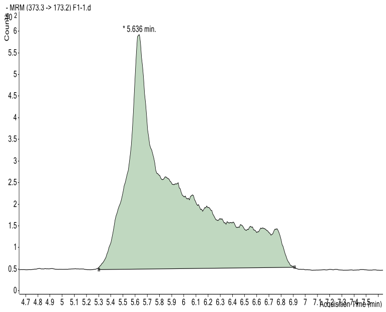	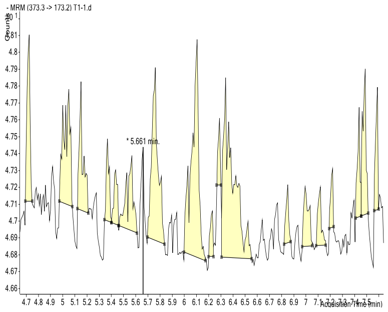
	2	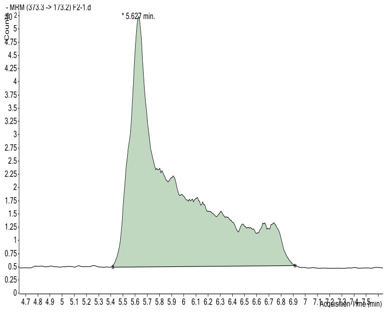	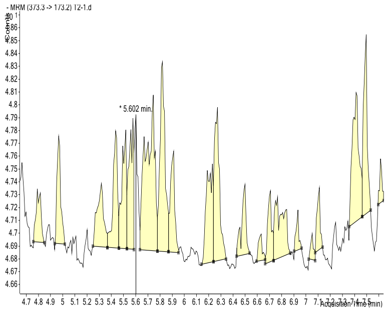
	5	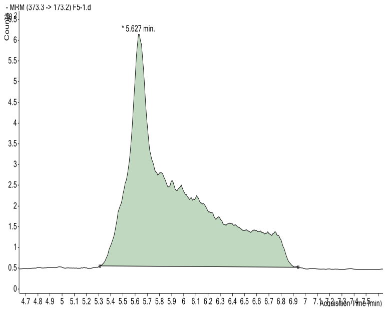	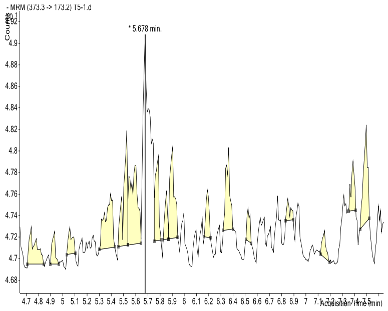
	8	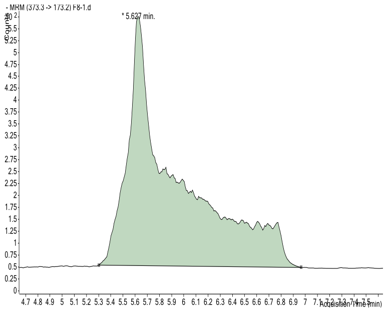	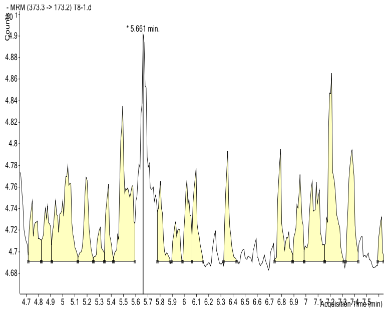
d6-20-HETE	1	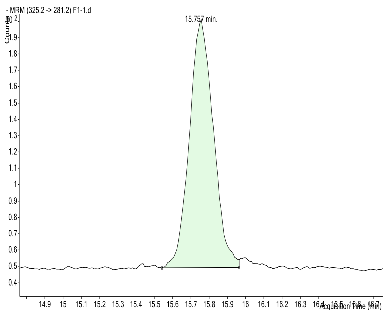	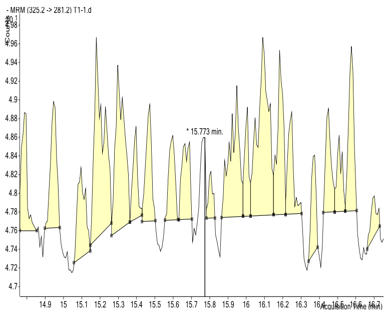

Surrogate	Oil volume (µL)	Polar lipid fraction	Neutral lipid fraction
	2	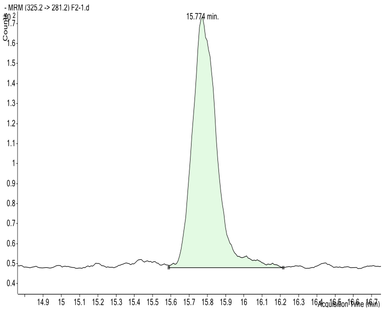	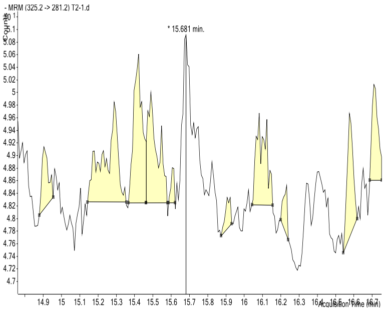
	5	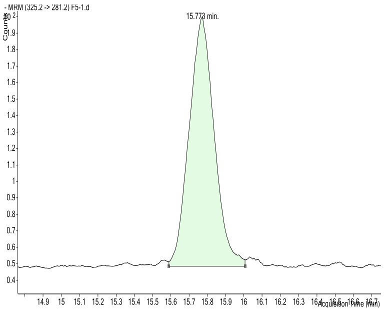	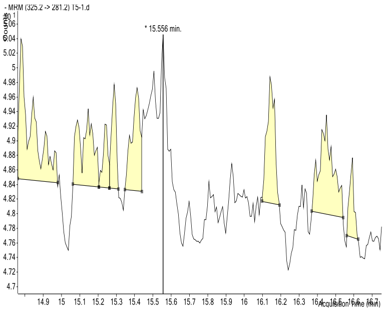
	8	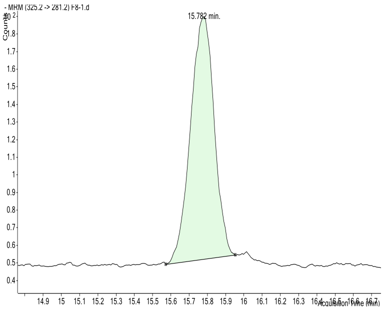	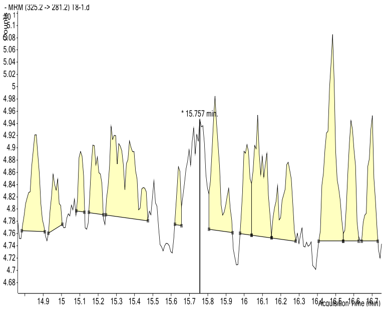
d8-5-HETE	1	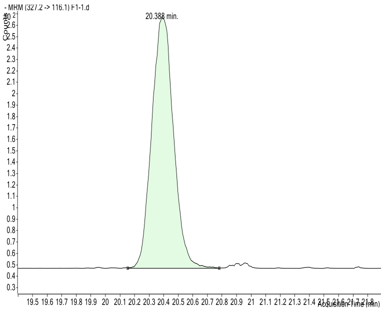	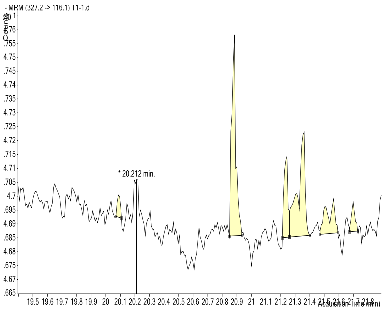
	2	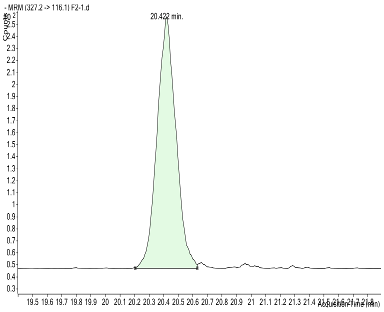	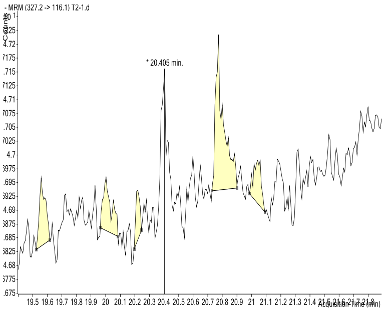

Surrogate	Oil volume (µL)	Polar lipid fraction	Neutral lipid fraction
	5	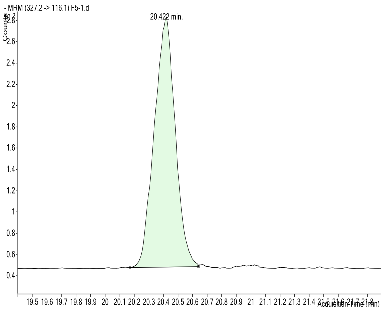	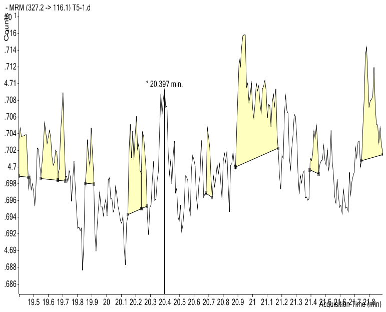
	8	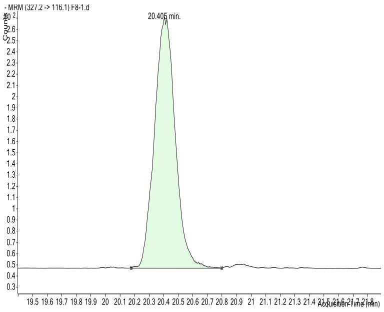	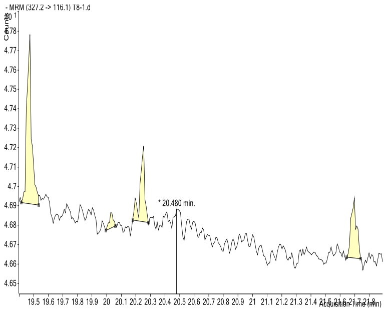

Supplementary Table 6. Retention time of the main fatty acids in soybean oil, as well as the internal standard used to quantify fatty acids.

Code	Name	RT (min)
	**Main fatty acids in soybean oil**	
C16:0	Palmitic Acid/Hexadecanoic Acid	14.7
C18:0	Stearic Acid/Octadecanoic Acid	17.2
C18:1 CIS	Oleic Acid	17.4
C18:2 n-6	Linoleic Acid (LA)	18.0
C18:3 n-3	linolenic acid (ALA)	18.9
	**Internal standard**	
C17:0	Margaric acid/Heptadecanoic acid	15.9

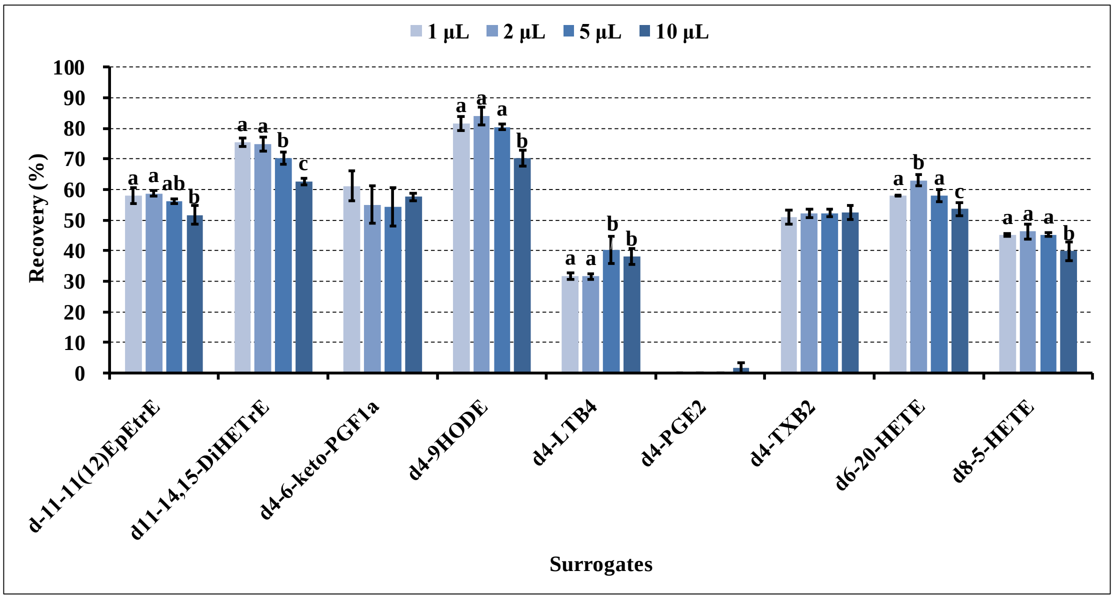

Supplementary Figure 1. Surrogate standard recovery from 1, 2, 5 and 10 µL of soybean oil following hydrolysis with sodium carbonate. Values are expressed at mean ± standard deviation (SD) of n=3 per oil volume. Data were analyzed by one-way ANOVA and Tukey's post-hoc test. Different letters within each surrogate are significantly different ($p < 0.05$).

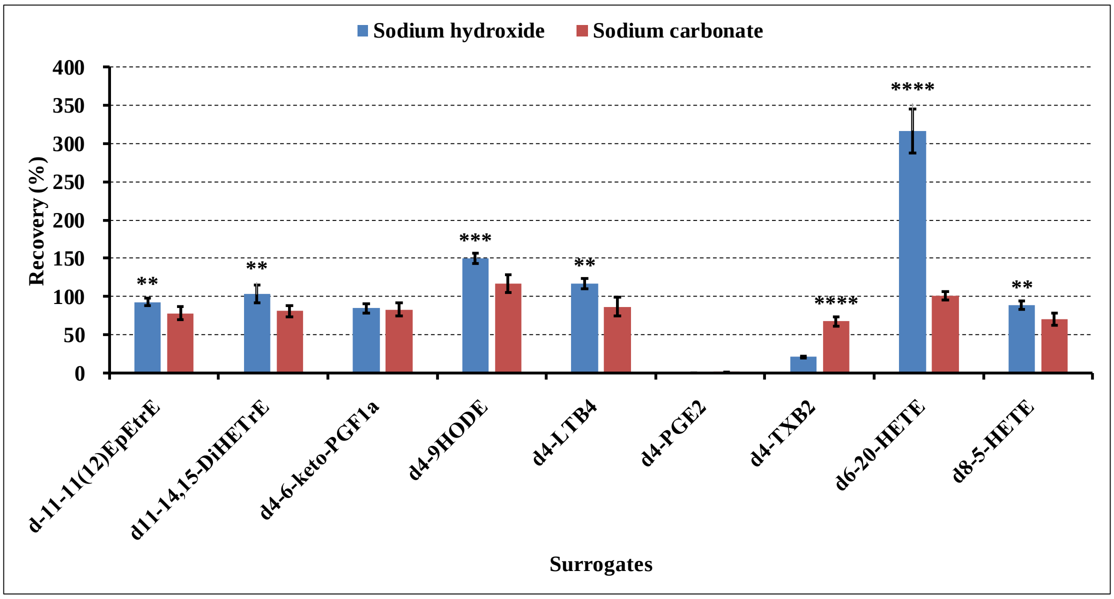

Supplementary Figure 2. Surrogate standard recovery following hydrolysis of 1 µL soybean oil with sodium hydroxide (NaOH) or sodium carbonate (Na_2CO_3). Values are expressed at mean ± standard deviation (SD) of n=5 per base condition. Data were analyzed by one-way ANOVA and Tukey's post-hoc test. Different letters within each surrogate are significantly different. * p <0.05; ** p <0.01; *** p <0.001; ****p <0.0001.

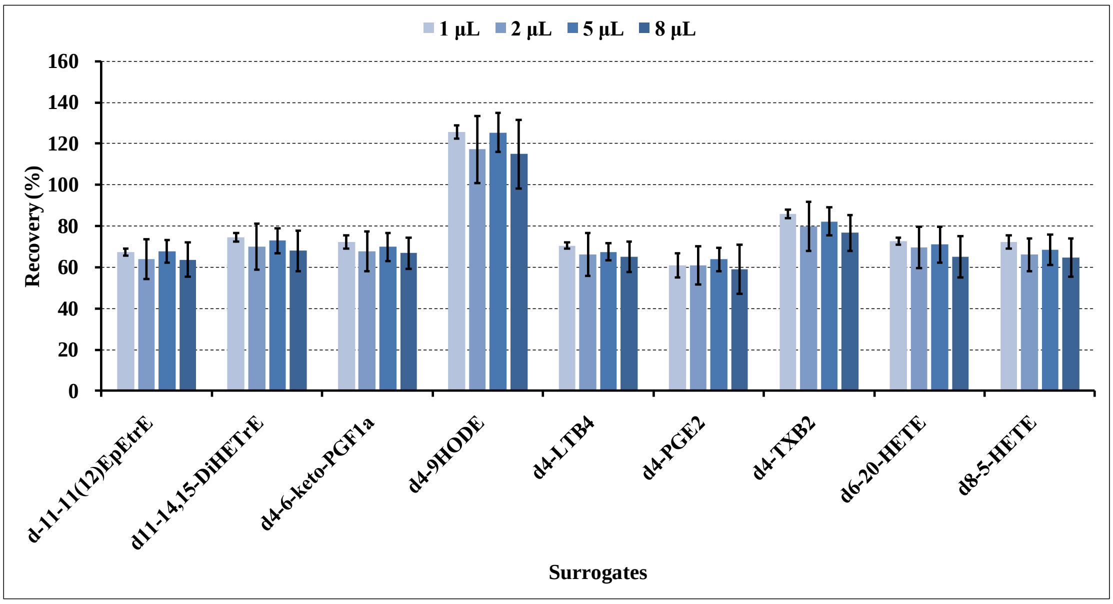

Supplementary Figure 3. Surrogate standard recovery following separation of free oxylipins from esterified oxylipins in 1, 2, 4 and 8 µL oil. Values are expressed at mean ± standard deviation (SD) of n=3 per oil volume. One-way ANOVA followed by Tukey's post-hoc test revealed no significant differences amongst the groups.

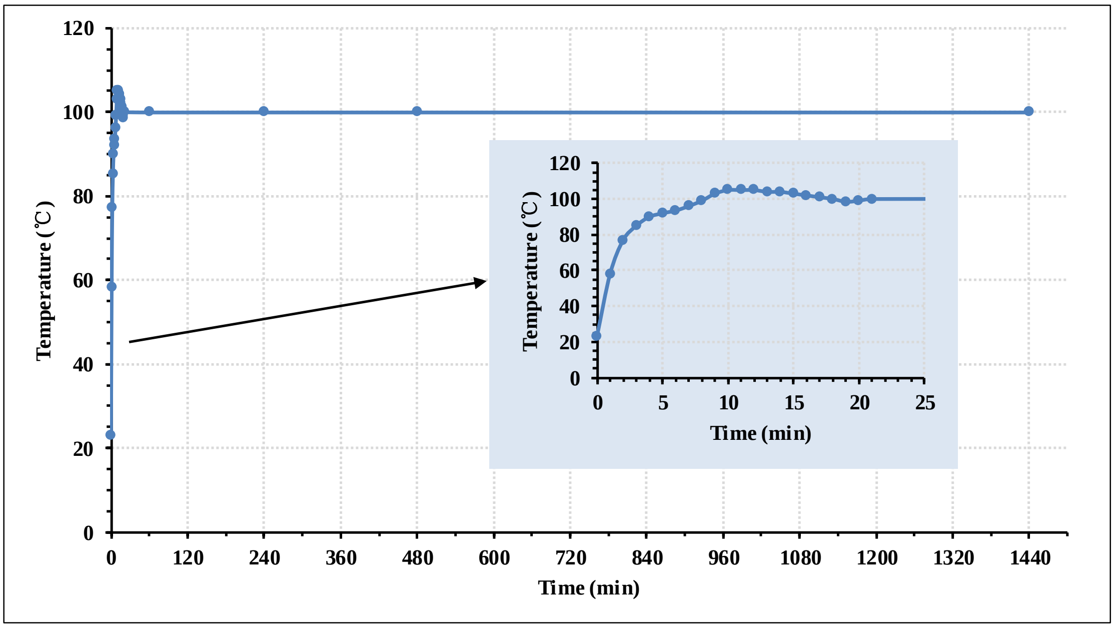

Supplementary Figure 4. Change in temperature over time of soybean oil heated at 100°C for 24 h (n=1 ; this is the sample in the 6th tube, next to 5 others sampled for oxylipin and fatty acid analysis throughout the experiment).

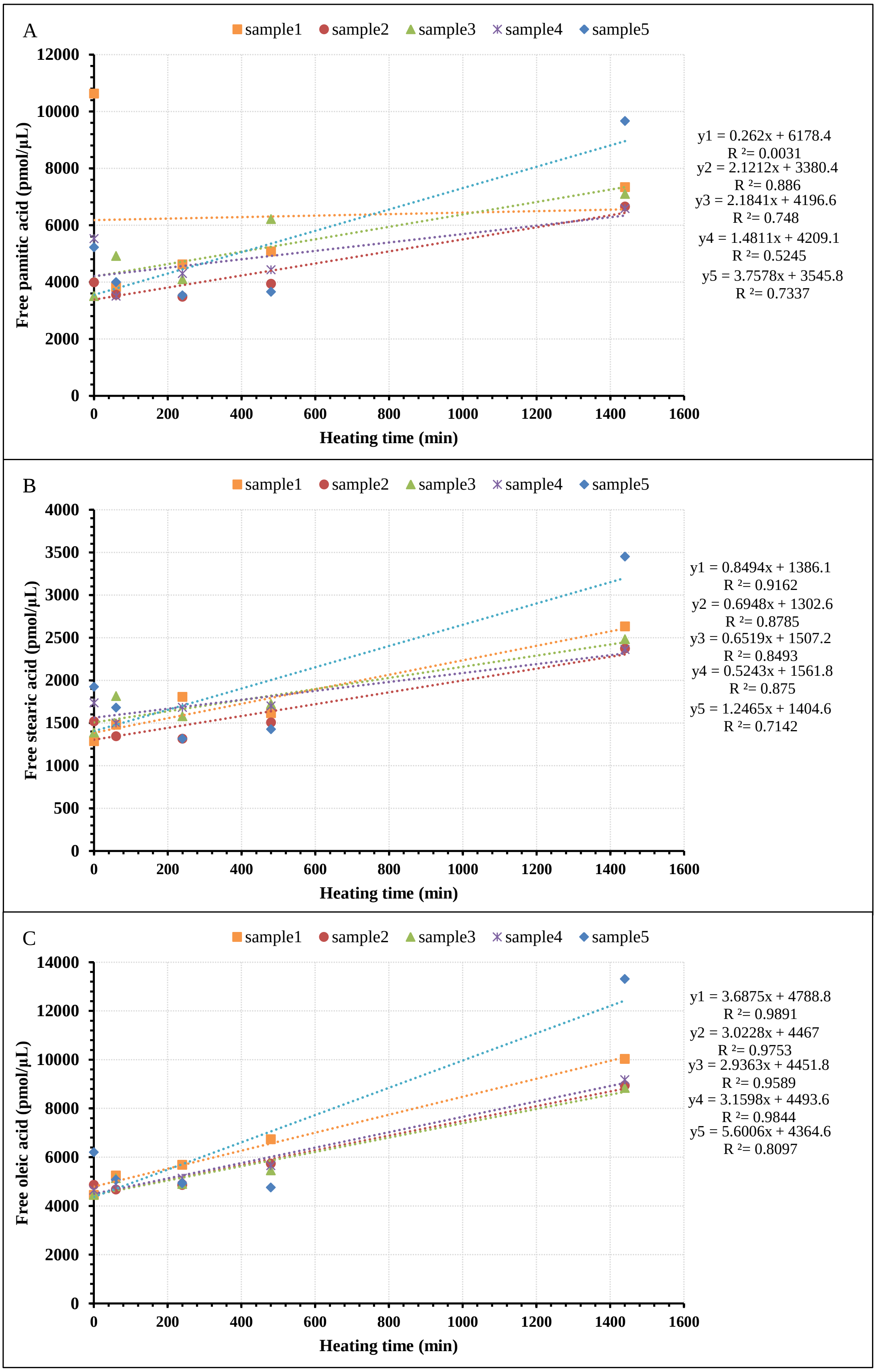
A
sample1 sample2 sample3 sample4 sample5
Free pamitic acid (pmol/μL)
Heating time (min)
y1 = 0.262x + 6178.4
R ²= 0.0031
y2 = 2.1212x + 3380.4
R ²= 0.886
y3 = 2.1841x + 4196.6
R ²= 0.748
y4 = 1.4811x + 4209.1
R ²= 0.5245
y5 = 3.7578x + 3545.8
R ²= 0.7337
B
sample1 sample2 sample3 sample4 sample5
Free stearic acid (pmol/μL)
Heating time (min)
y1 = 0.8494x + 1386.1
R ²= 0.9162
y2 = 0.6948x + 1302.6
R ²= 0.8785
y3 = 0.6519x + 1507.2
R ²= 0.8493
y4 = 0.5243x + 1561.8
R ²= 0.875
y5 = 1.2465x + 1404.6
R ²= 0.7142
C
sample1 sample2 sample3 sample4 sample5
Free oleic acid (pmol/μL)
Heating time (min)
y1 = 3.6875x + 4788.8
R ²= 0.9891
y2 = 3.0228x + 4467
R ²= 0.9753
y3 = 2.9363x + 4451.8
R ²= 0.9589
y4 = 3.1598x + 4493.6
R ²= 0.9844
y5 = 5.6006x + 4364.6
R ²= 0.8097

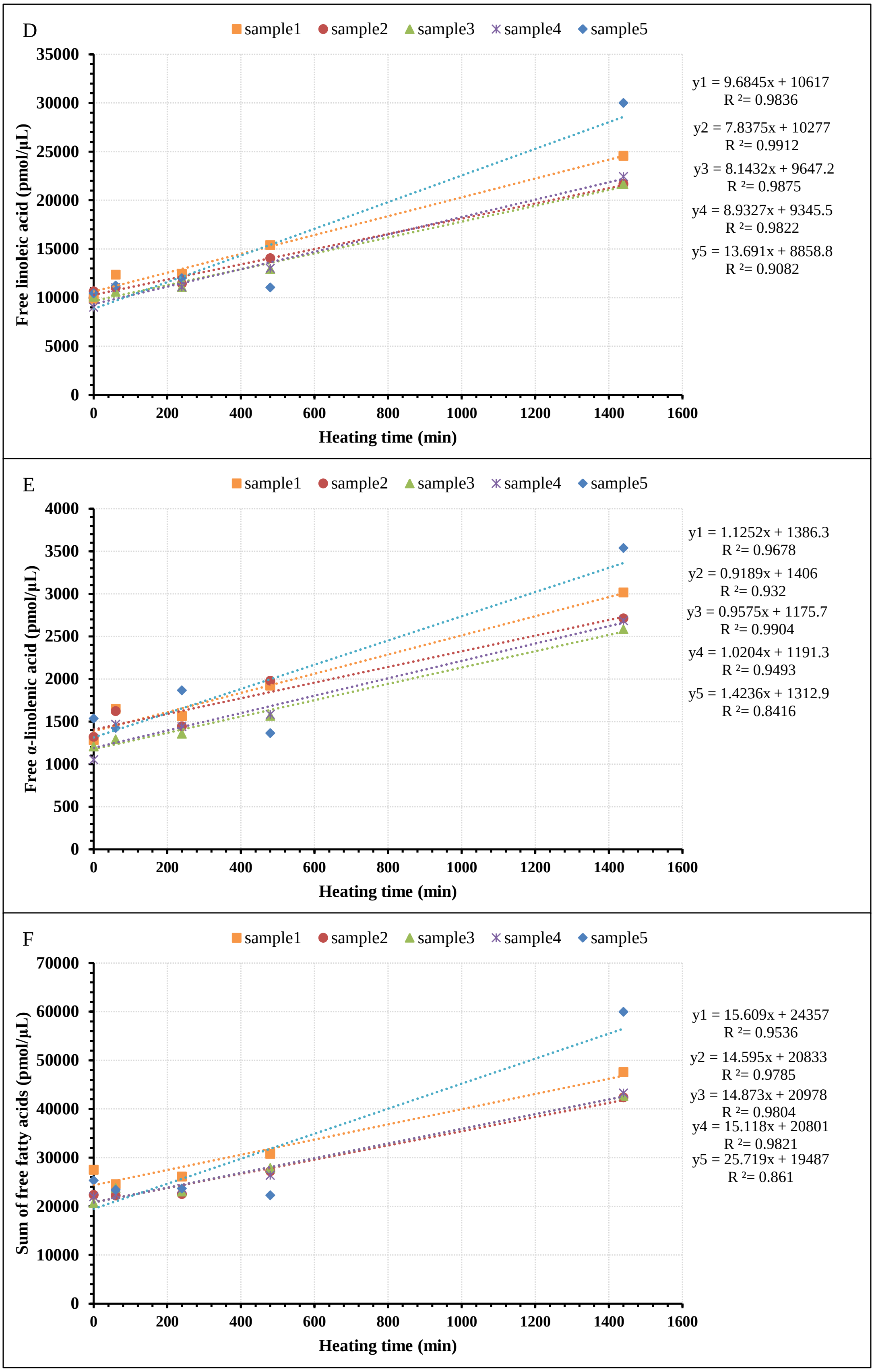

Supplementary Figure 5. Linear fit of the Concentration-Time plot of free pamitic acid (A), stearic acid (B), oleic acid (C), linoleic acid (LA; D), α-linolenic acid (ALA; E) and sum of free fatty acids (F) in soybean oil heated for 24 h (n=5 per time-point).

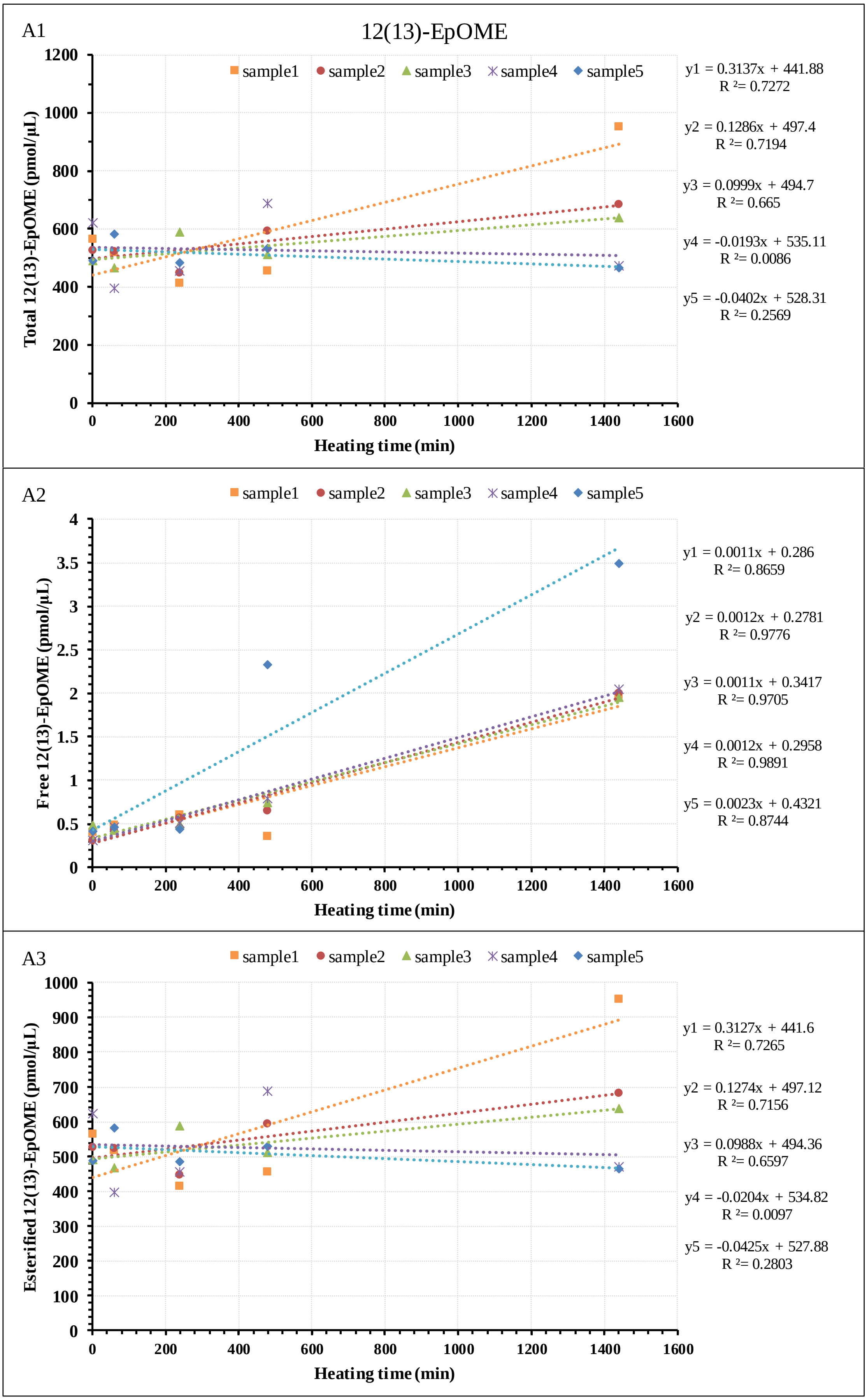
A1
12(13)-EpOME
Total 12(13)-EpOME (pmol/μL)
sample1 sample2 sample3 sample4 sample5
y1 = 0.3137x + 441.88
R ²= 0.7272
y2 = 0.1286x + 497.4
R ²= 0.7194
y3 = 0.0999x + 494.7
R ²= 0.665
y4 = -0.0193x + 535.11
R ²= 0.0086
y5 = -0.0402x + 528.31
R ²= 0.2569
Heating time (min)
A2
Free 12(13)-EpOME (pmol/μL)
sample1 sample2 sample3 sample4 sample5
y1 = 0.0011x + 0.286
R ²= 0.8659
y2 = 0.0012x + 0.2781
R ²= 0.9776
y3 = 0.0011x + 0.3417
R ²= 0.9705
y4 = 0.0012x + 0.2958
R ²= 0.9891
y5 = 0.0023x + 0.4321
R ²= 0.8744
Heating time (min)
A3
Esterified 12(13)-EpOME (pmol/μL)
sample1 sample2 sample3 sample4 sample5
y1 = 0.3127x + 441.6
R ²= 0.7265
y2 = 0.1274x + 497.12
R ²= 0.7156
y3 = 0.0988x + 494.36
R ²= 0.6597
y4 = -0.0204x + 534.82
R ²= 0.0097
y5 = -0.0425x + 527.88
R ²= 0.2803
Heating time (min)

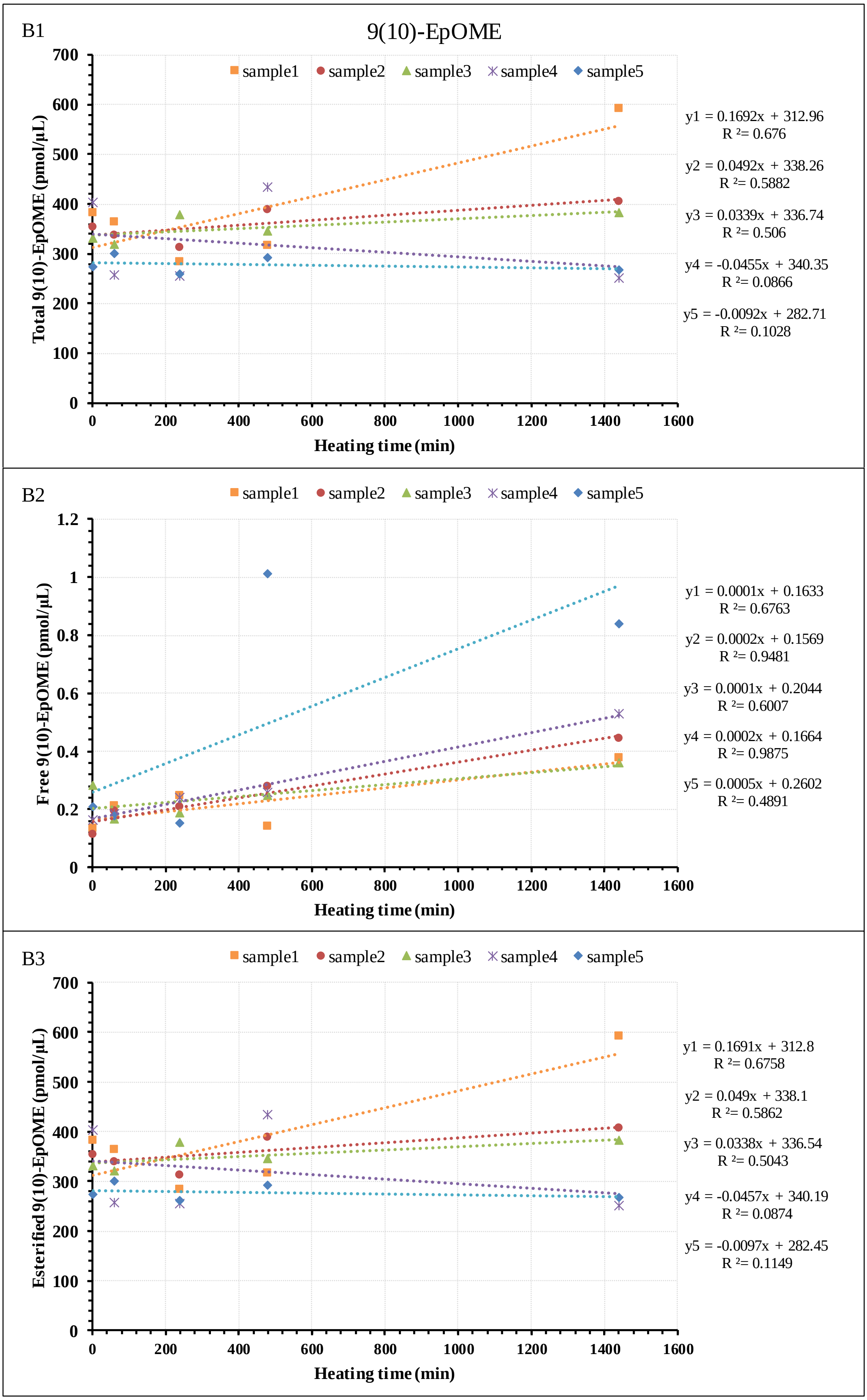
B1
9(10)-EpOME
sample1 sample2 sample3 sample4 sample5
Total 9(10)-EpOME (pmol/μL)
Heating time (min)
y1 = 0.1692x + 312.96
R ²= 0.676
y2 = 0.0492x + 338.26
R ²= 0.5882
y3 = 0.0339x + 336.74
R ²= 0.506
y4 = -0.0455x + 340.35
R ²= 0.0866
y5 = -0.0092x + 282.71
R ²= 0.1028
B2
sample1 sample2 sample3 sample4 sample5
Free 9(10)-EpOME (pmol/μL)
Heating time (min)
y1 = 0.0001x + 0.1633
R ²= 0.6763
y2 = 0.0002x + 0.1569
R ²= 0.9481
y3 = 0.0001x + 0.2044
R ²= 0.6007
y4 = 0.0002x + 0.1664
R ²= 0.9875
y5 = 0.0005x + 0.2602
R ²= 0.4891
B3
sample1 sample2 sample3 sample4 sample5
Esterified 9(10)-EpOME (pmol/μL)
Heating time (min)
y1 = 0.1691x + 312.8
R ²= 0.6758
y2 = 0.049x + 338.1
R ²= 0.5862
y3 = 0.0338x + 336.54
R ²= 0.5043
y4 = -0.0457x + 340.19
R ²= 0.0874
y5 = -0.0097x + 282.45
R ²= 0.1149

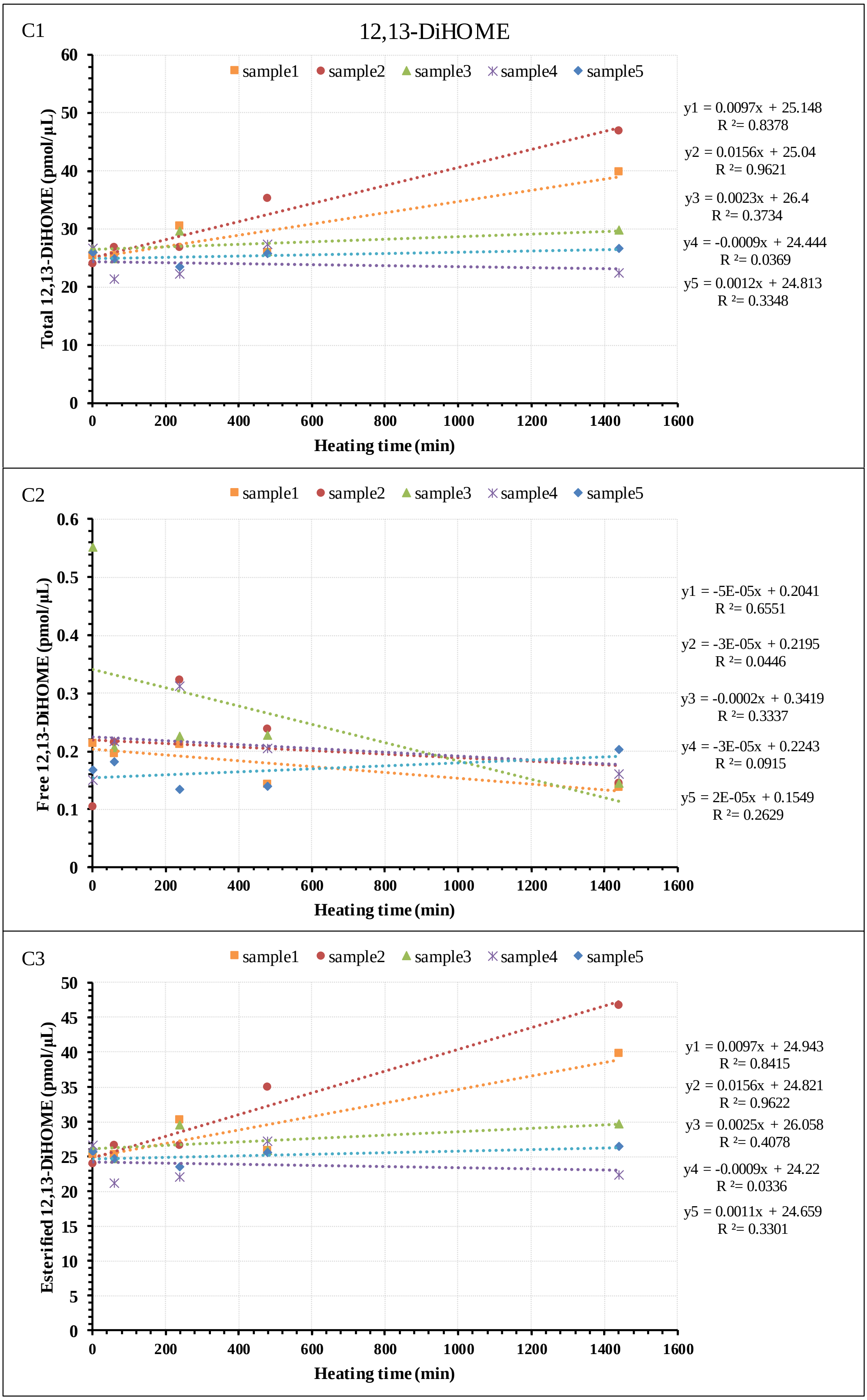

C1
12,13-DiHOME
sample1 sample2 sample3 sample4 sample5
Total 12,13-DiHOME (pmol/μL)
0 10 20 30 40 50 60
0 200 400 600 800 1000 1200 1400 1600
Heating time (min)
y1 = 0.0097x + 25.148
R ²= 0.8378
y2 = 0.0156x + 25.04
R ²= 0.9621
y3 = 0.0023x + 26.4
R ²= 0.3734
y4 = -0.0009x + 24.444
R ²= 0.0369
y5 = 0.0012x + 24.813
R ²= 0.3348
C2
sample1 sample2 sample3 sample4 sample5
Free 12,13-DiHOME (pmol/μL)
0 0.1 0.2 0.3 0.4 0.5 0.6
0 200 400 600 800 1000 1200 1400 1600
Heating time (min)
y1 = -5E-05x + 0.2041
R ²= 0.6551
y2 = -3E-05x + 0.2195
R ²= 0.0446
y3 = -0.0002x + 0.3419
R ²= 0.3337
y4 = -3E-05x + 0.2243
R ²= 0.0915
y5 = 2E-05x + 0.1549
R ²= 0.2629
C3
sample1 sample2 sample3 sample4 sample5
Esterified 12,13-DiHOME (pmol/μL)
0 5 10 15 20 25 30 35 40 45 50
0 200 400 600 800 1000 1200 1400 1600
Heating time (min)
y1 = 0.0097x + 24.943
R ²= 0.8415
y2 = 0.0156x + 24.821
R ²= 0.9622
y3 = 0.0025x + 26.058
R ²= 0.4078
y4 = -0.0009x + 24.22
R ²= 0.0336
y5 = 0.0011x + 24.659
R ²= 0.3301

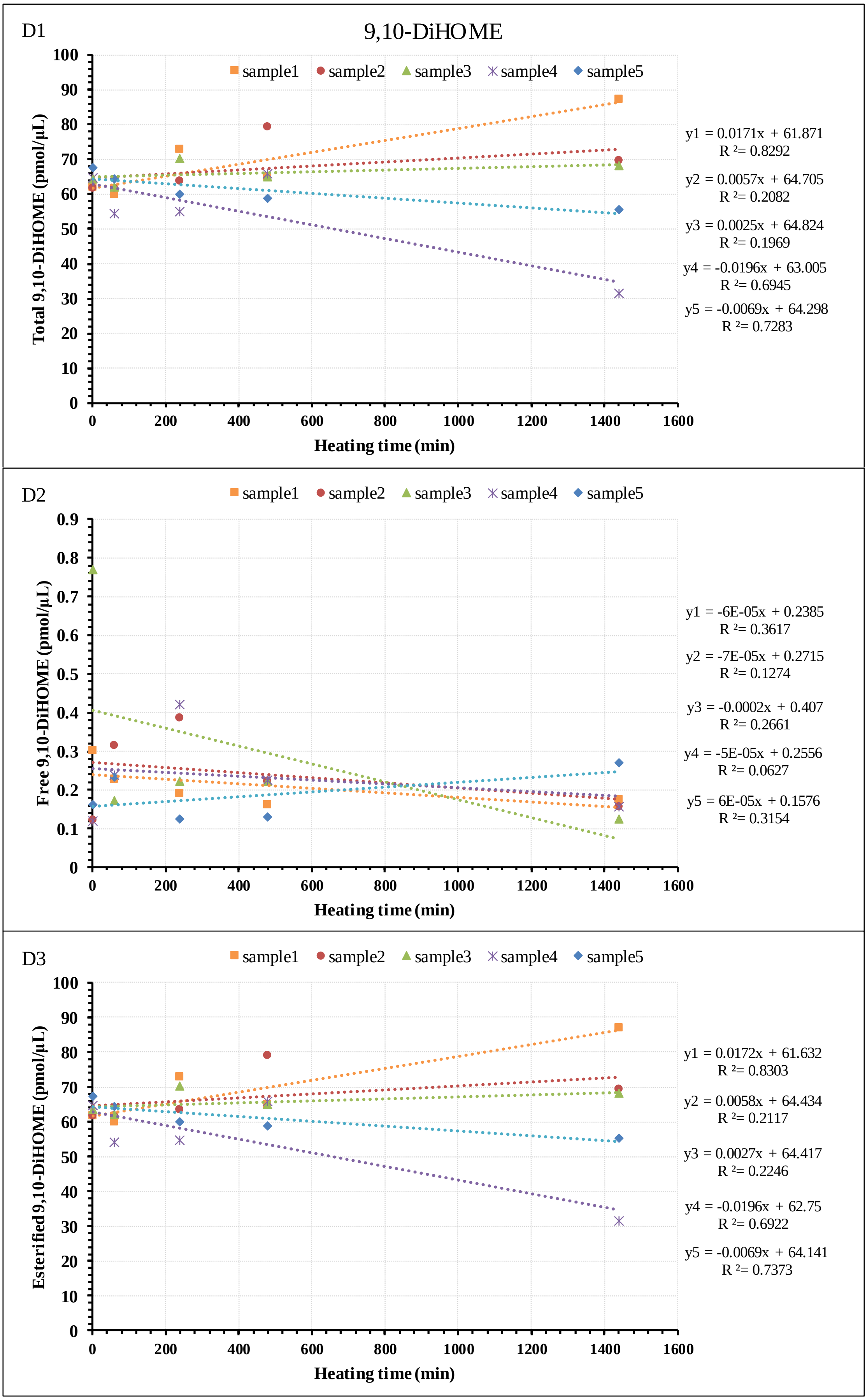

D1
9,10-DiHOME
sample1 sample2 sample3 sample4 sample5
Total 9,10-DiHOME (pmol/µL)
Heating time (min)
y1 = 0.0171x + 61.871
R ²= 0.8292
y2 = 0.0057x + 64.705
R ²= 0.2082
y3 = 0.0025x + 64.824
R ²= 0.1969
y4 = -0.0196x + 63.005
R ²= 0.6945
y5 = -0.0069x + 64.298
R ²= 0.7283
D2
sample1 sample2 sample3 sample4 sample5
Free 9,10-DiHOME (pmol/µL)
Heating time (min)
y1 = -6E-05x + 0.2385
R ²= 0.3617
y2 = -7E-05x + 0.2715
R ²= 0.1274
y3 = -0.0002x + 0.407
R ²= 0.2661
y4 = -5E-05x + 0.2556
R ²= 0.0627
y5 = 6E-05x + 0.1576
R ²= 0.3154
D3
sample1 sample2 sample3 sample4 sample5
Esterified 9,10-DiHOME (pmol/µL)
Heating time (min)
y1 = 0.0172x + 61.632
R ²= 0.8303
y2 = 0.0058x + 64.434
R ²= 0.2117
y3 = 0.0027x + 64.417
R ²= 0.2246
y4 = -0.0196x + 62.75
R ²= 0.6922
y5 = -0.0069x + 64.141
R ²= 0.7373

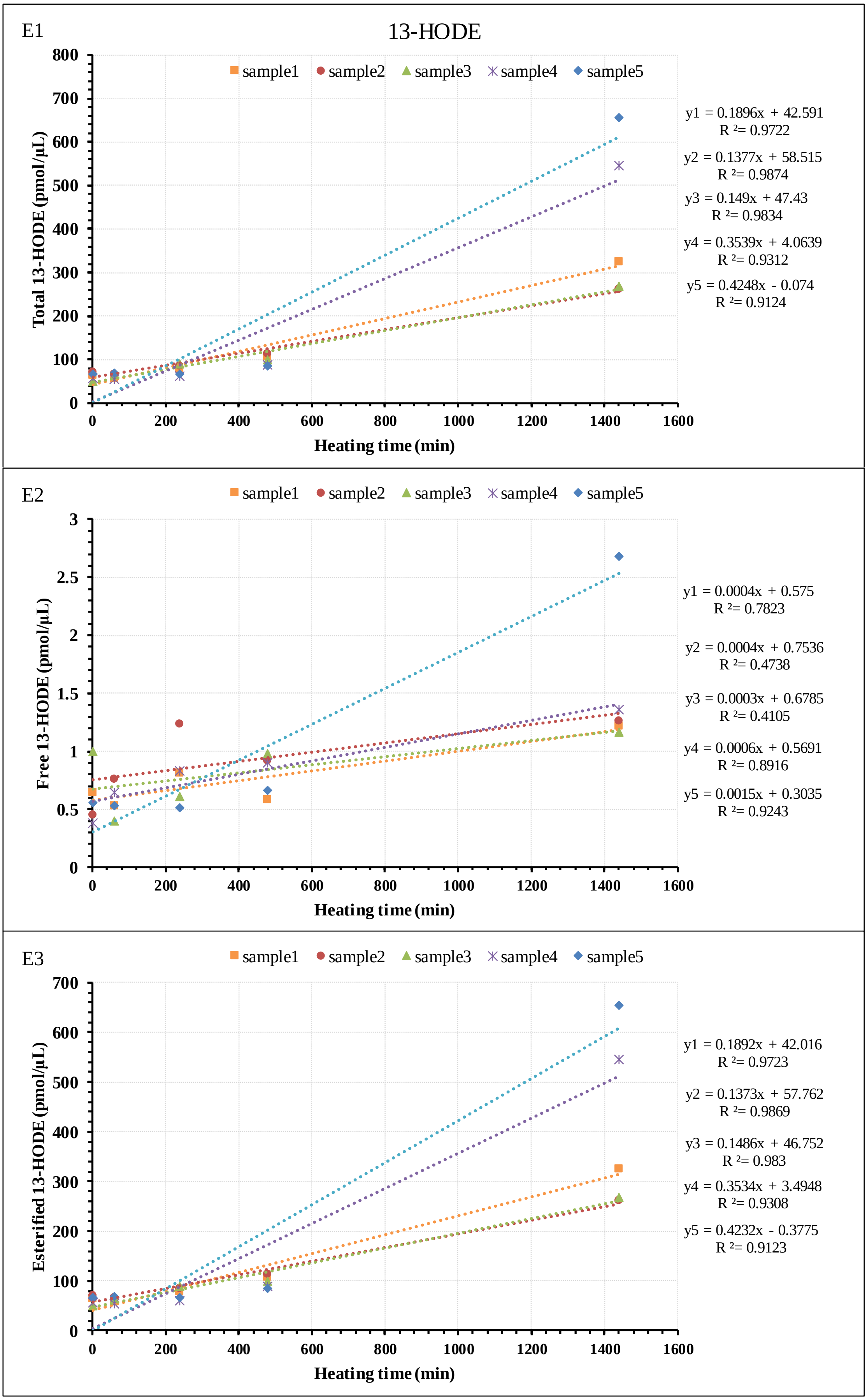
E1
13-HODE
sample1 sample2 sample3 sample4 sample5
Total 13-HODE (pmol/µL)
Heating time (min)
y1 = 0.1896x + 42.591
R ²= 0.9722
y2 = 0.1377x + 58.515
R ²= 0.9874
y3 = 0.149x + 47.43
R ²= 0.9834
y4 = 0.3539x + 4.0639
R ²= 0.9312
y5 = 0.4248x - 0.074
R ²= 0.9124
E2
Free 13-HODE (pmol/µL)
Heating time (min)
y1 = 0.0004x + 0.575
R ²= 0.7823
y2 = 0.0004x + 0.7536
R ²= 0.4738
y3 = 0.0003x + 0.6785
R ²= 0.4105
y4 = 0.0006x + 0.5691
R ²= 0.8916
y5 = 0.0015x + 0.3035
R ²= 0.9243
E3
Esterified 13-HODE (pmol/µL)
Heating time (min)
y1 = 0.1892x + 42.016
R ²= 0.9723
y2 = 0.1373x + 57.762
R ²= 0.9869
y3 = 0.1486x + 46.752
R ²= 0.983
y4 = 0.3534x + 3.4948
R ²= 0.9308
y5 = 0.4232x - 0.3775
R ²= 0.9123

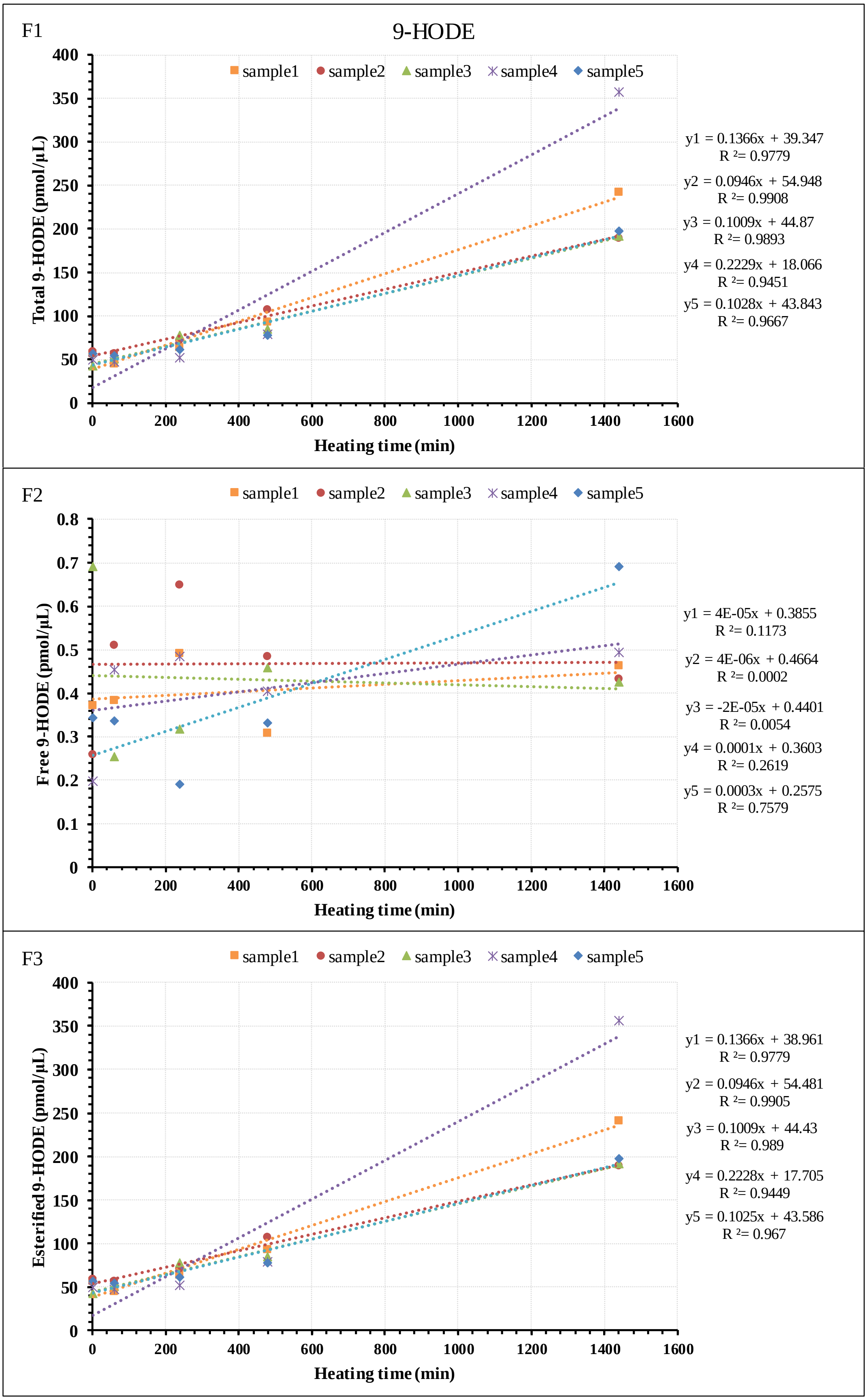
F1
9-HODE
sample1
sample2
sample3
sample4
sample5
Total 9-HODE (pmol/μL)
Heating time (min)
y1 = 0.1366x + 39.347
R ²= 0.9779
y2 = 0.0946x + 54.948
R ²= 0.9908
y3 = 0.1009x + 44.87
R ²= 0.9893
y4 = 0.2229x + 18.066
R ²= 0.9451
y5 = 0.1028x + 43.843
R ²= 0.9667
F2
Free 9-HODE (pmol/μL)
Heating time (min)
y1 = 4E-05x + 0.3855
R ²= 0.1173
y2 = 4E-06x + 0.4664
R ²= 0.0002
y3 = -2E-05x + 0.4401
R ²= 0.0054
y4 = 0.0001x + 0.3603
R ²= 0.2619
y5 = 0.0003x + 0.2575
R ²= 0.7579
F3
Esterified 9-HODE (pmol/μL)
Heating time (min)
y1 = 0.1366x + 38.961
R ²= 0.9779
y2 = 0.0946x + 54.481
R ²= 0.9905
y3 = 0.1009x + 44.43
R ²= 0.989
y4 = 0.2228x + 17.705
R ²= 0.9449
y5 = 0.1025x + 43.586
R ²= 0.967

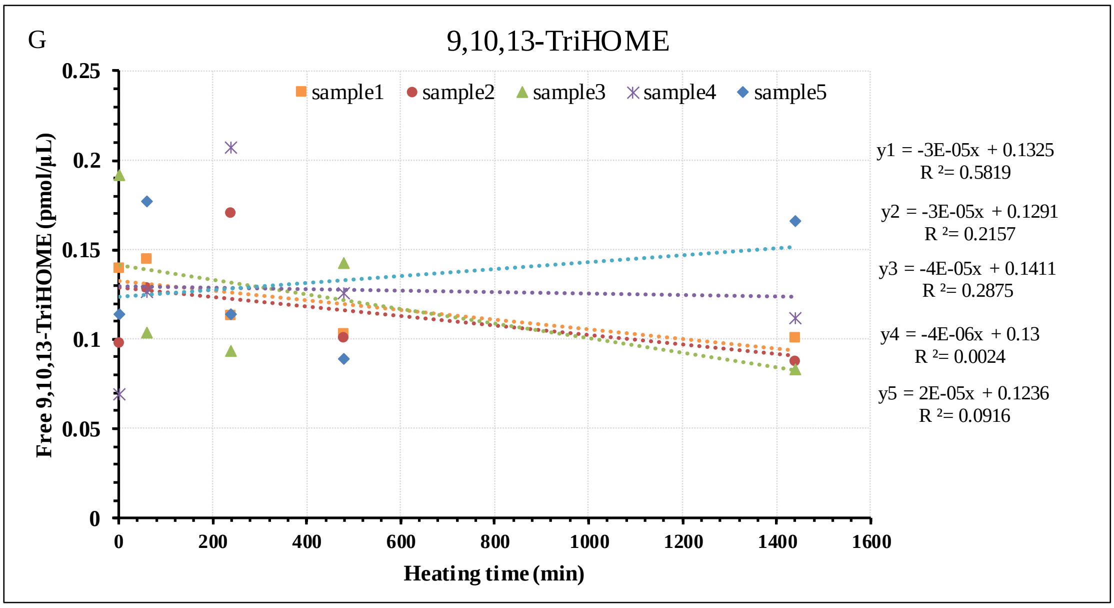
G
9,10,13-TriHOME
sample1 sample2 sample3 sample4 sample5
Free 9,10,13-TriHOME (pmol/µL)
0.25
0.2
0.15
0.1
0.05
0
0 200 400 600 800 1000 1200 1400 1600
Heating time (min)
y1 = -3E-05x + 0.1325
R ²= 0.5819
y2 = -3E-05x + 0.1291
R ²= 0.2157
y3 = -4E-05x + 0.1411
R ²= 0.2875
y4 = -4E-06x + 0.13
R ²= 0.0024
y5 = 2E-05x + 0.1236
R ²= 0.0916

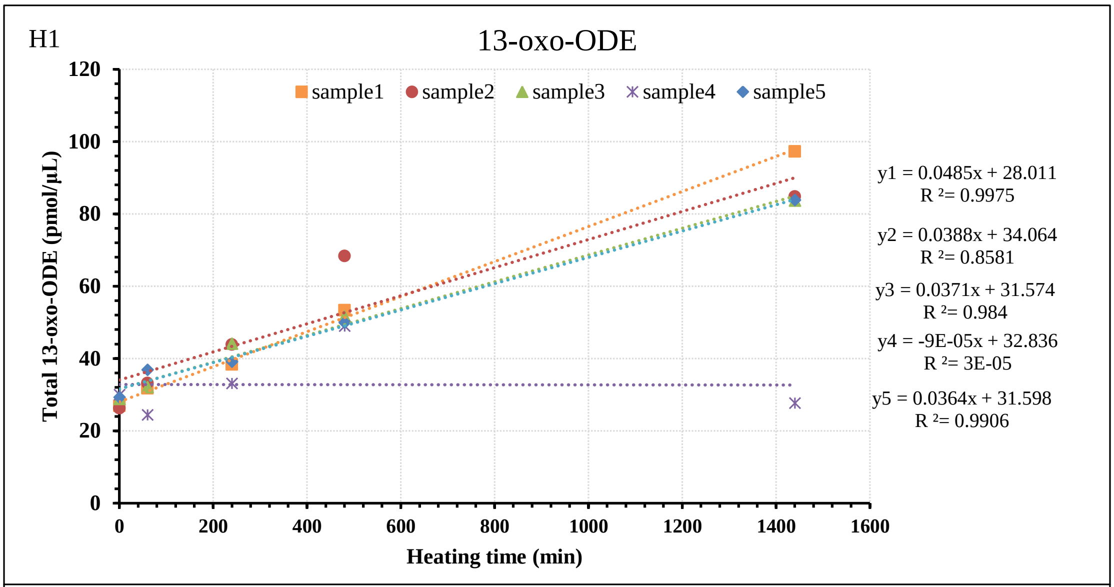
H1
13-oxo-ODE
sample1 sample2 sample3 sample4 sample5
Total 13-oxo-ODE (pmol/µL)
120
100
80
60
40
20
0
0 200 400 600 800 1000 1200 1400 1600
Heating time (min)
y1 = 0.0485x + 28.011
R ²= 0.9975
y2 = 0.0388x + 34.064
R ²= 0.8581
y3 = 0.0371x + 31.574
R ²= 0.984
y4 = -9E-05x + 32.836
R ²= 3E-05
y5 = 0.0364x + 31.598
R ²= 0.9906

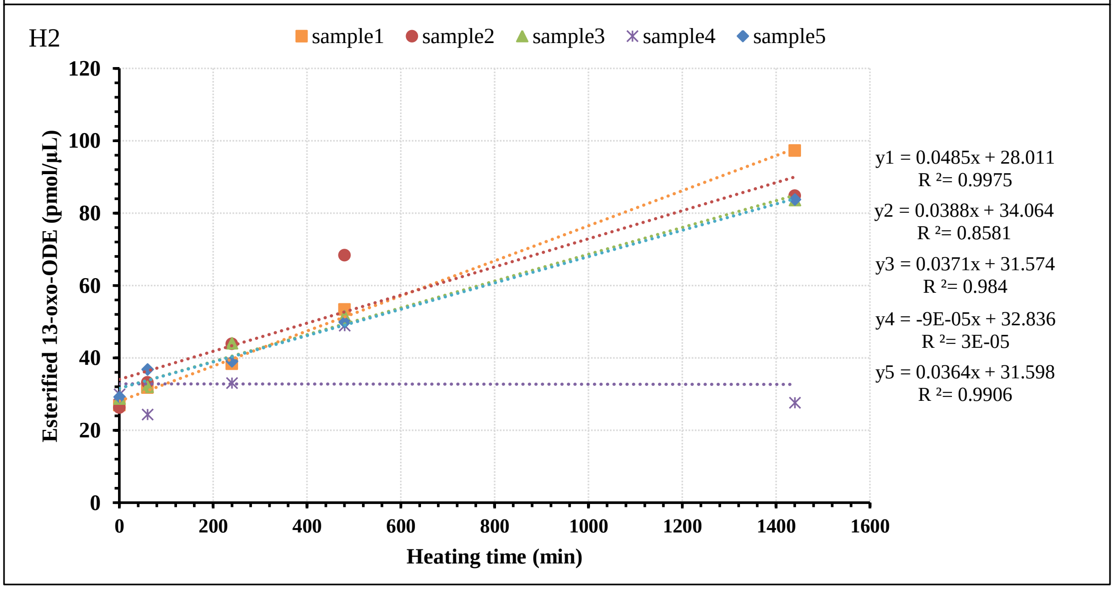
H2
sample1 sample2 sample3 sample4 sample5
Esterified 13-oxo-ODE (pmol/µL)
120
100
80
60
40
20
0
0 200 400 600 800 1000 1200 1400 1600
Heating time (min)
y1 = 0.0485x + 28.011
R ²= 0.9975
y2 = 0.0388x + 34.064
R ²= 0.8581
y3 = 0.0371x + 31.574
R ²= 0.984
y4 = -9E-05x + 32.836
R ²= 3E-05
y5 = 0.0364x + 31.598
R ²= 0.9906

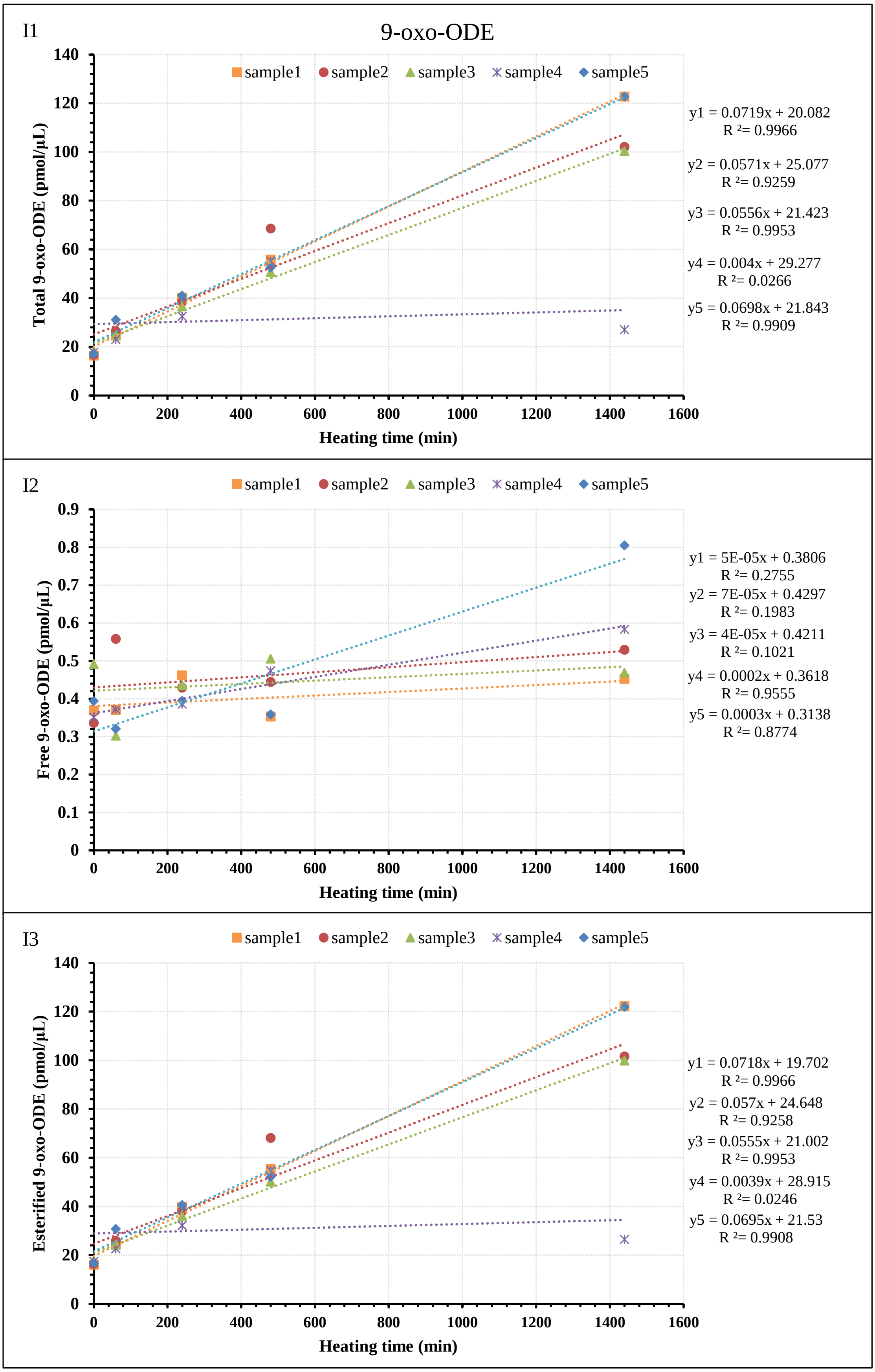
I1
9-oxo-ODE
sample1 sample2 sample3 sample4 sample5
Total 9-oxo-ODE (pmol/μL)
Heating time (min)
y1 = 0.0719x + 20.082
R ²= 0.9966
y2 = 0.0571x + 25.077
R ²= 0.9259
y3 = 0.0556x + 21.423
R ²= 0.9953
y4 = 0.004x + 29.277
R ²= 0.0266
y5 = 0.0698x + 21.843
R ²= 0.9909
I2
sample1 sample2 sample3 sample4 sample5
Free 9-oxo-ODE (pmol/μL)
Heating time (min)
y1 = 5E-05x + 0.3806
R ²= 0.2755
y2 = 7E-05x + 0.4297
R ²= 0.1983
y3 = 4E-05x + 0.4211
R ²= 0.1021
y4 = 0.0002x + 0.3618
R ²= 0.9555
y5 = 0.0003x + 0.3138
R ²= 0.8774
I3
sample1 sample2 sample3 sample4 sample5
Esterified 9-oxo-ODE (pmol/μL)
Heating time (min)
y1 = 0.0718x + 19.702
R ²= 0.9966
y2 = 0.057x + 24.648
R ²= 0.9258
y3 = 0.0555x + 21.002
R ²= 0.9953
y4 = 0.0039x + 28.915
R ²= 0.0246
y5 = 0.0695x + 21.53
R ²= 0.9908

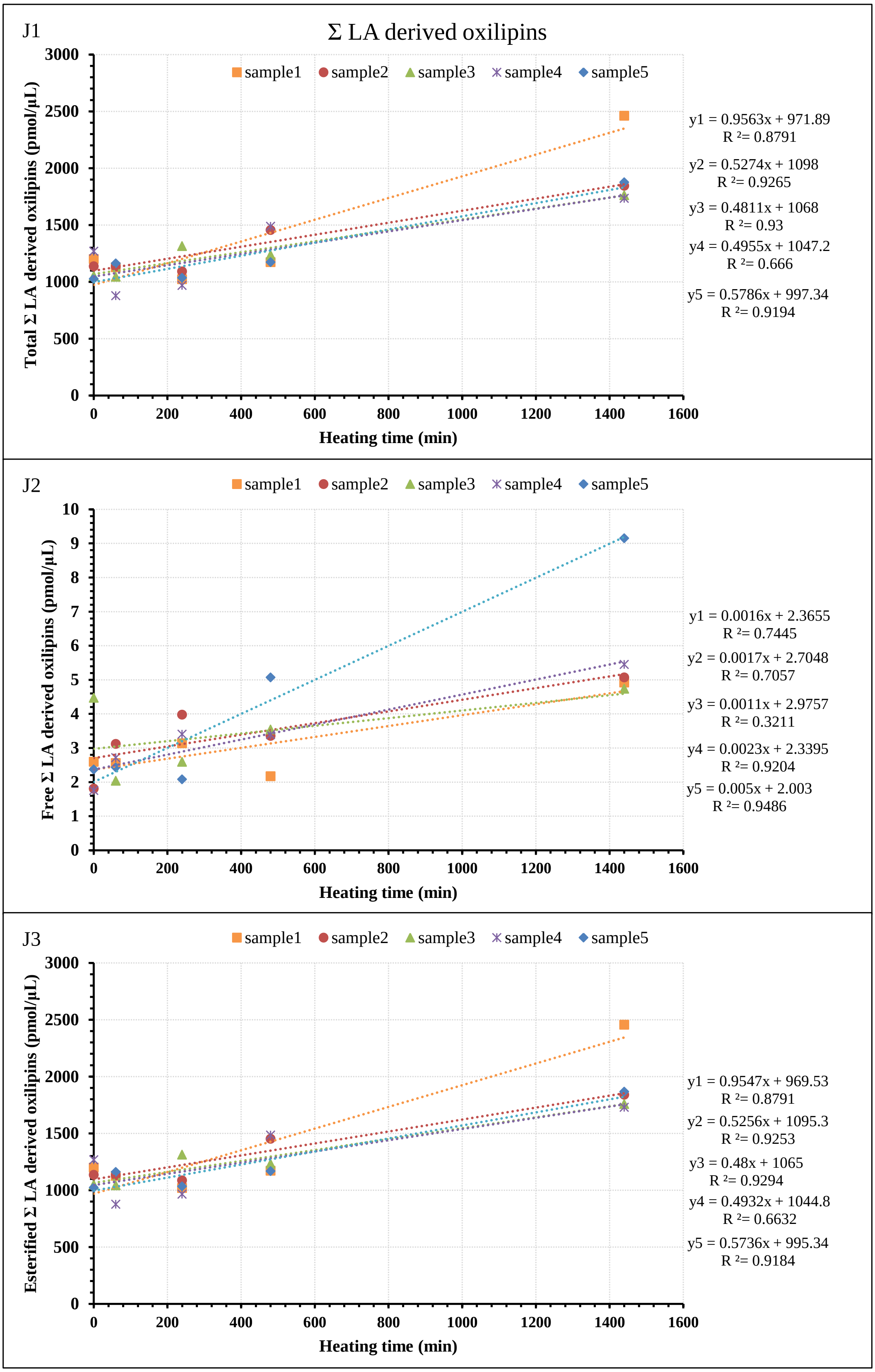
J1
Σ LA derived oxilipins
Total Σ LA derived oxilipins (pmol/μL)
sample1 sample2 sample3 sample4 sample5
y1 = 0.9563x + 971.89
R ²= 0.8791
y2 = 0.5274x + 1098
R ²= 0.9265
y3 = 0.4811x + 1068
R ²= 0.93
y4 = 0.4955x + 1047.2
R ²= 0.666
y5 = 0.5786x + 997.34
R ²= 0.9194
Heating time (min)
J2
Free Σ LA derived oxilipins (pmol/μL)
sample1 sample2 sample3 sample4 sample5
y1 = 0.0016x + 2.3655
R ²= 0.7445
y2 = 0.0017x + 2.7048
R ²= 0.7057
y3 = 0.0011x + 2.9757
R ²= 0.3211
y4 = 0.0023x + 2.3395
R ²= 0.9204
y5 = 0.005x + 2.003
R ²= 0.9486
Heating time (min)
J3
Esterified Σ LA derived oxilipins (pmol/μL)
sample1 sample2 sample3 sample4 sample5
y1 = 0.9547x + 969.53
R ²= 0.8791
y2 = 0.5256x + 1095.3
R ²= 0.9253
y3 = 0.48x + 1065
R ²= 0.9294
y4 = 0.4932x + 1044.8
R ²= 0.6632
y5 = 0.5736x + 995.34
R ²= 0.9184
Heating time (min)

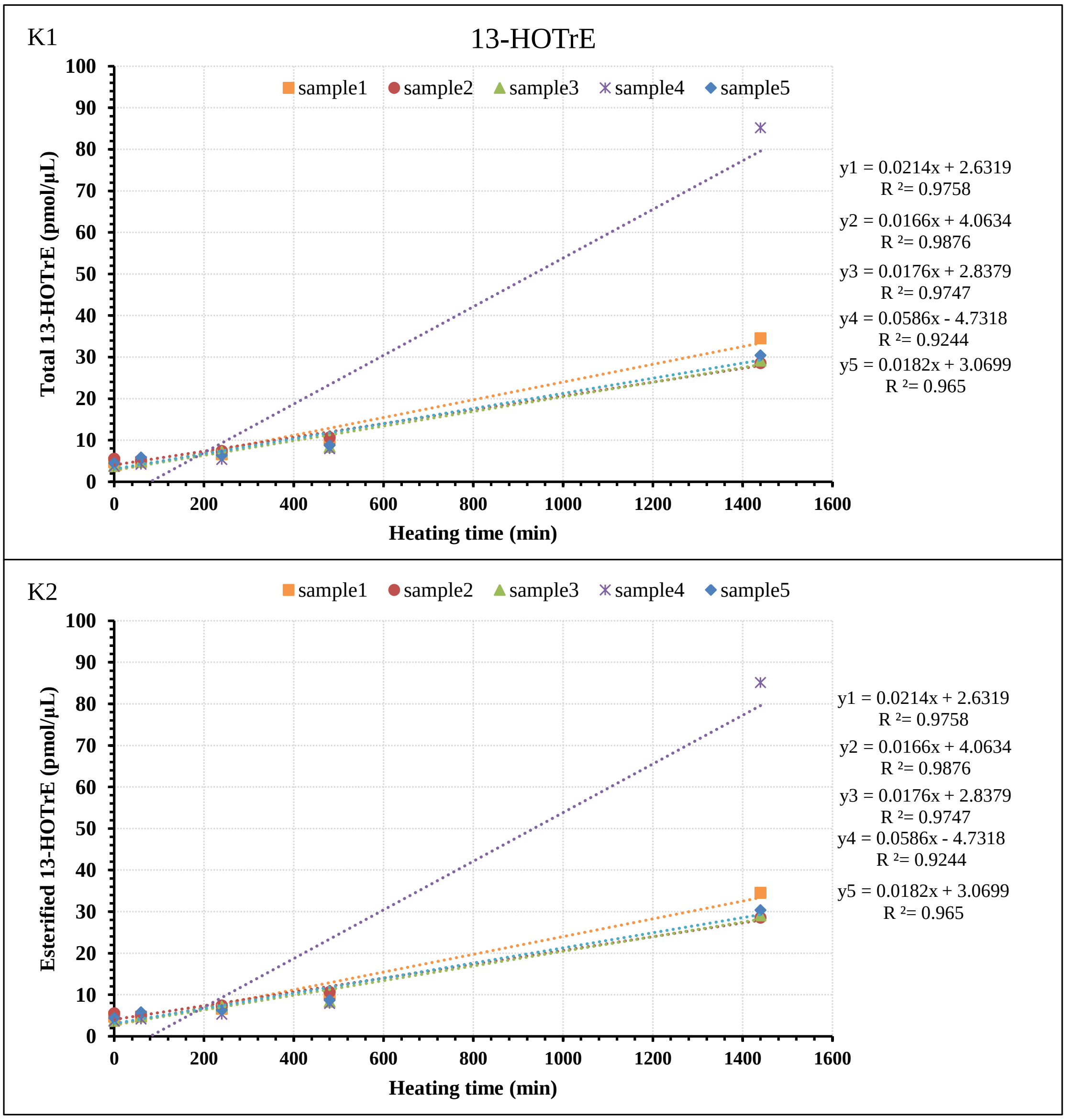
K1
13-HOTrE
sample1 sample2 sample3 sample4 sample5
Total 13-HOTrE (pmol/µL)
Heating time (min)
y1 = 0.0214x + 2.6319
R ²= 0.9758
y2 = 0.0166x + 4.0634
R ²= 0.9876
y3 = 0.0176x + 2.8379
R ²= 0.9747
y4 = 0.0586x - 4.7318
R ²= 0.9244
y5 = 0.0182x + 3.0699
R ²= 0.965
K2
sample1 sample2 sample3 sample4 sample5
Esterified 13-HOTrE (pmol/µL)
Heating time (min)
y1 = 0.0214x + 2.6319
R ²= 0.9758
y2 = 0.0166x + 4.0634
R ²= 0.9876
y3 = 0.0176x + 2.8379
R ²= 0.9747
y4 = 0.0586x - 4.7318
R ²= 0.9244
y5 = 0.0182x + 3.0699
R ²= 0.965

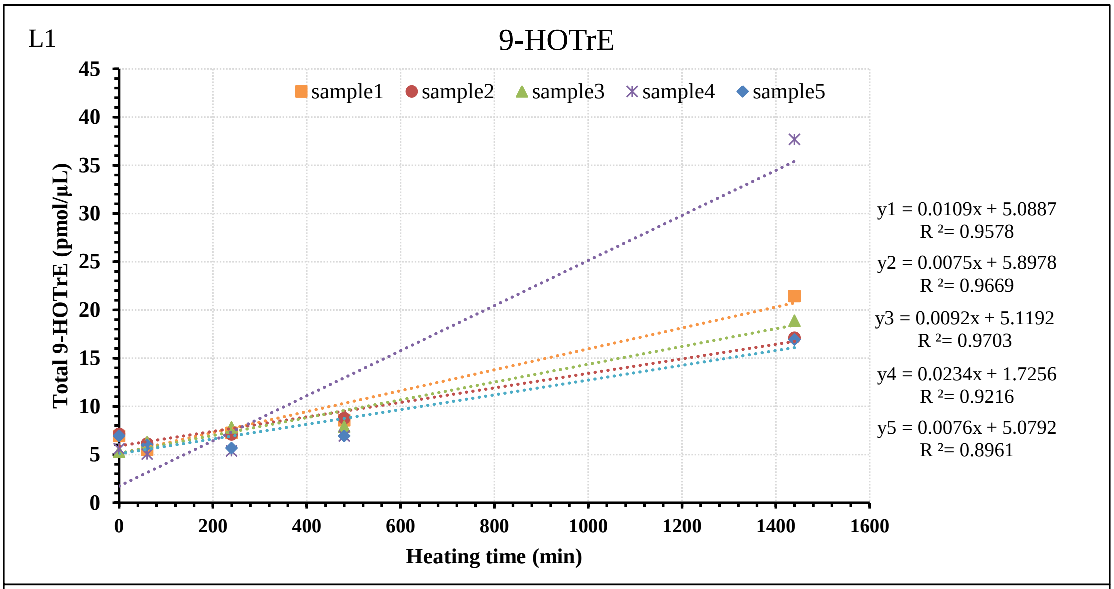
L1
9-HOTrE
sample1 sample2 sample3 sample4 sample5
Total 9-HOTrE (pmol/μL)
Heating time (min)
0 200 400 600 800 1000 1200 1400 1600
0 5 10 15 20 25 30 35 40 45
y1 = 0.0109x + 5.0887
R ²= 0.9578
y2 = 0.0075x + 5.8978
R ²= 0.9669
y3 = 0.0092x + 5.1192
R ²= 0.9703
y4 = 0.0234x + 1.7256
R ²= 0.9216
y5 = 0.0076x + 5.0792
R ²= 0.8961

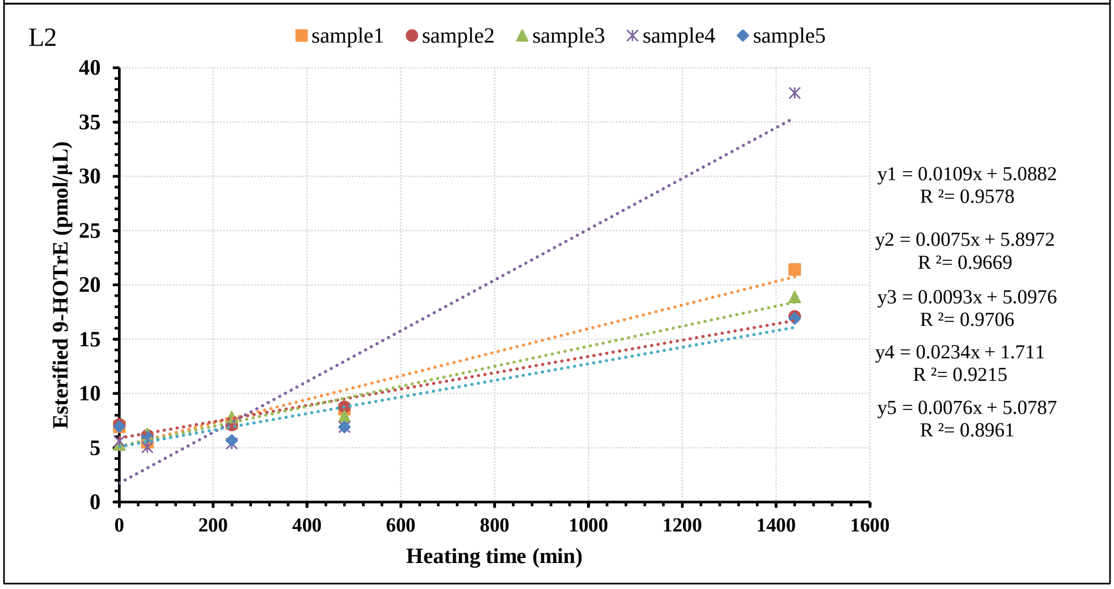
L2
sample1 sample2 sample3 sample4 sample5
Esterified 9-HOTrE (pmol/μL)
Heating time (min)
0 200 400 600 800 1000 1200 1400 1600
0 5 10 15 20 25 30 35 40
y1 = 0.0109x + 5.0882
R ²= 0.9578
y2 = 0.0075x + 5.8972
R ²= 0.9669
y3 = 0.0093x + 5.0976
R ²= 0.9706
y4 = 0.0234x + 1.711
R ²= 0.9215
y5 = 0.0076x + 5.0787
R ²= 0.8961

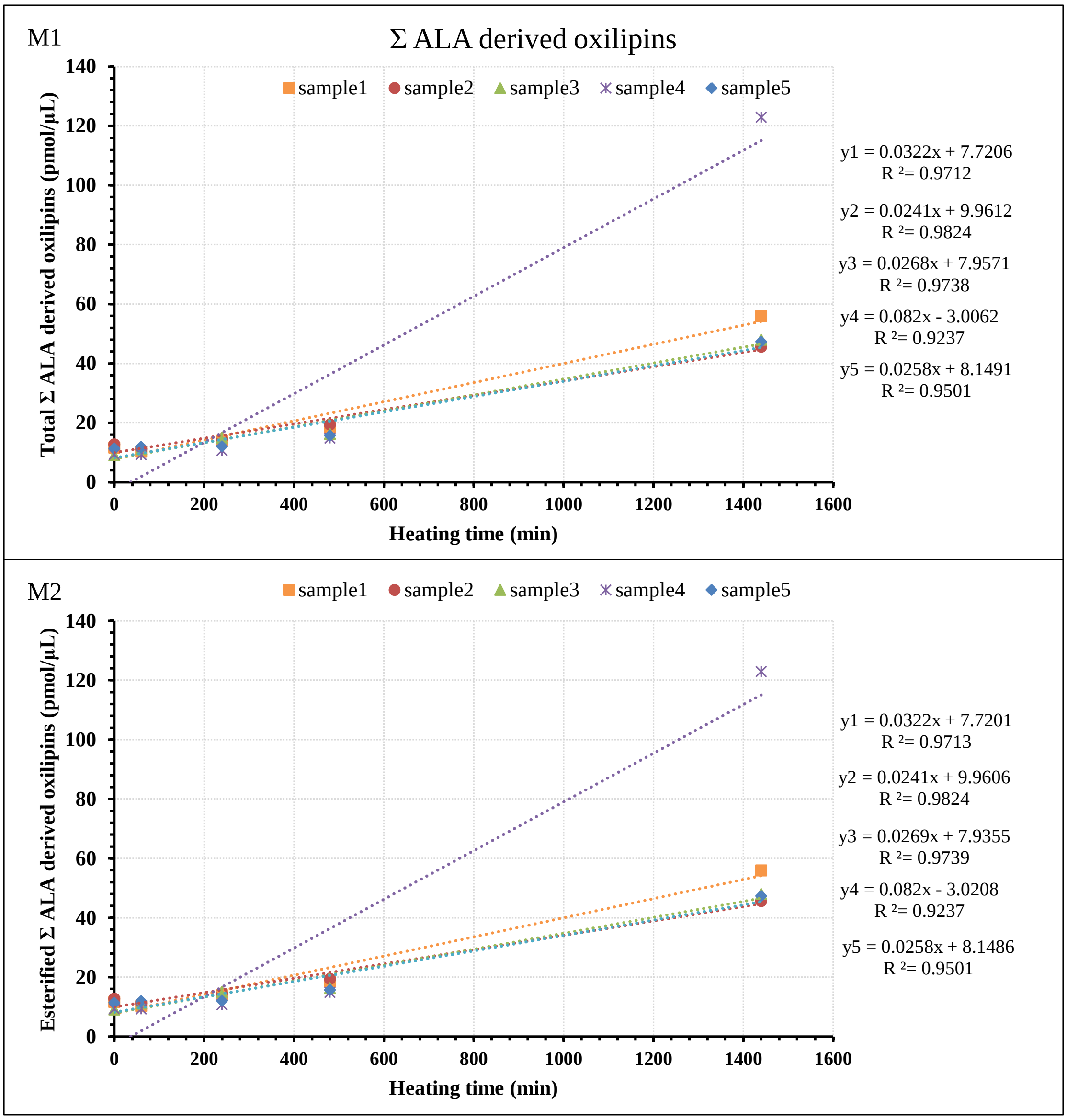

Supplementary Figure 6. Linear regression fit for Concentration-Time plots of total, free and esterified oxylipins formed in soybean oil heated for 24 h. 12(13)-EpOME (total/free/esterified, A1/A2/A3); 9(10)-EpOME (total/free/esterified, B1/B2/B3); 12,13-DiHOME (total/free/esterified, C1/C2/C3); 9,10-DiHOME (total/free/esterified, D1/D2/D3); 13-HODE (total/free/esterified, E1/E2/E3); 9-HODE (total/free/esterified, F1/F2/F3); 9,10,13-TriHOME (free, G); 13-oxo-ODE (total/esterified, H1/H2); 9-oxo-ODE (total/free/esterified, I1/I2/I3); Σ LA-derived oxylipins (total/free/esterified, J1/J2/J3); 13-HOTrE (total/esterified, K1/K2); 9-HOTrE (total/ esterified, L1/L2); Σ ALA-derived oxylipins (total/esterified, M1/M2). (n=5 per time point)

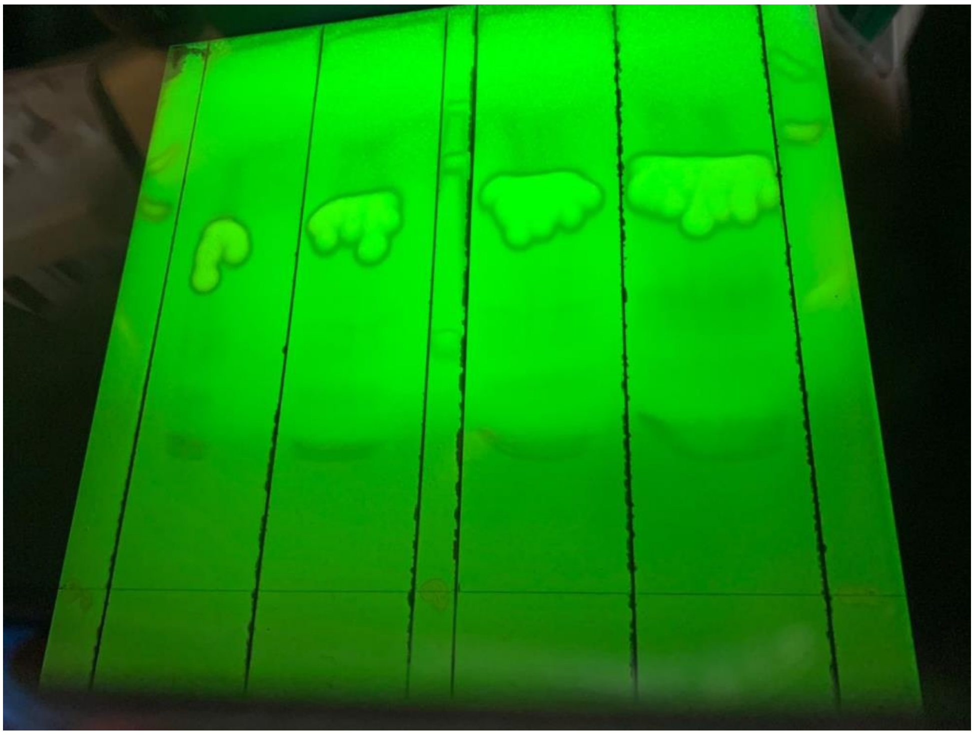

Supplementary Figure 7. Thin layer chromatography (TLC) of various soybean oil volumes, to determine the optimal amount that separates free fatty acids. Sample from the left to right lane are: TLC standard mix, 1 μL soybean oil, 2 μL soybean oil, TLC standard mix, 3 μL soybean oil, 4 μL soybean oil, and TLC standard mix. The TLC standard mix contained phospholipids, cholesterol, FFAs, TAGs and cholesteryl esters. Phospholipids appear at the bottom, followed by cholesterol, FFAs, TAGs and cholesteryl esters.
